# Therapeutic Potential and Activity Modulation of the Protein Lysine Deacylase Sirtuin 5

**DOI:** 10.1021/acs.jmedchem.2c00687

**Published:** 2022-07-08

**Authors:** Francesco Fiorentino, Carola Castiello, Antonello Mai, Dante Rotili

**Affiliations:** † Department of Drug Chemistry and Technologies, Sapienza University of Rome, Piazzala Aldo Moro 5, Rome 00185, Italy; ‡ Pasteur Institute, Cenci-Bolognetti Foundation, Sapienza University of Rome, Piazzala Aldo Moro 5, Rome 00185, Italy

## Abstract

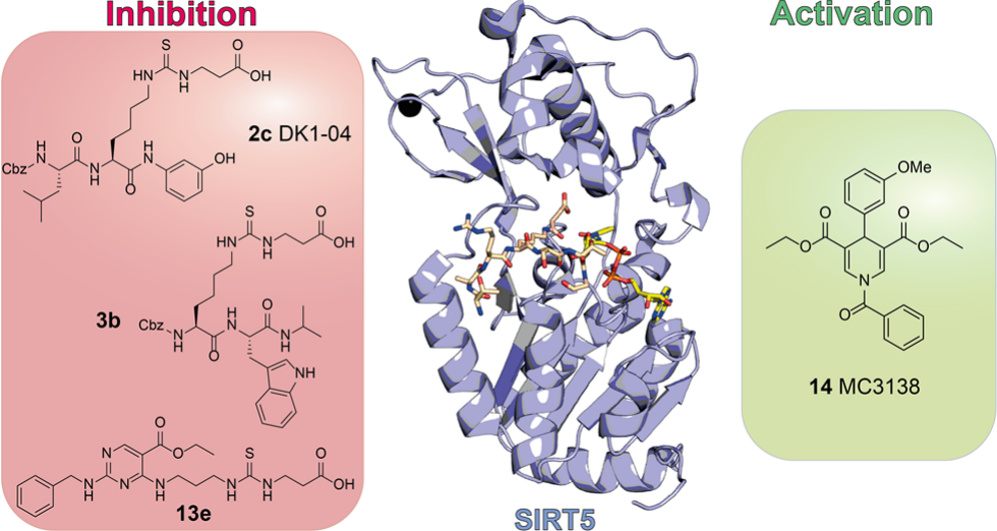

Sirtiun 5 (SIRT5) is a NAD^+^-dependent protein lysine deacylase primarily located in mitochondria. SIRT5 displays an affinity for negatively charged acyl groups and mainly catalyzes lysine deglutarylation, desuccinylation, and demalonylation while possessing weak deacetylase activity. SIRT5 substrates play crucial roles in metabolism and reactive oxygen species (ROS) detoxification, and SIRT5 activity is protective in neuronal and cardiac physiology. Moreover, SIRT5 exhibits a dichotomous role in cancer, acting as context-dependent tumor promoter or suppressor. Given its multifaceted activity, SIRT5 is a promising target in the design of activators or inhibitors that might act as therapeutics in many pathologies, including cancer, cardiovascular disorders, and neurodegeneration. To date, few cellular-active peptide-based SIRT5 inhibitors (SIRT5i) have been described, and potent and selective small-molecule SIRT5i have yet to be discovered. In this perspective, we provide an outline of SIRT5’s roles in different biological settings and describe SIRT5 modulators in terms of their mode of action, pharmacological activity, and structure–activity relationships.

## 1 Introduction

Following translation, proteins may undergo post-translational modifications (PTMs) on their amino acid side chains, expanding the spectrum of functions, stability, and subcellular localization. Most PTMs are dynamic, thereby enabling each protein to interchange between many functional states. In particular, the ε-*N*-lysine residues of proteins are subject to many PTMs, such as alkylation and acylation.^1^ Among these PTMs, lysine acetylation was initially identified on histone proteins in the 1960s and linked to transcriptional regulation.^2^ Subsequent studies identified the enzymes that catalyzed the transfer of acetyl groups to histones, called histone acetyltransferases (HATs),^3^ while the enzymes that catalyzed the removal of acetyl groups were named histone deacetylases (HDACs).^4−6^ Later investigations demonstrated that proteins other than histones^7^ may also undergo (de)acetylation, and recent studies have revealed other acyllysine modifications other than acetylation (e.g., lysines acylated with short-, medium-, and long-chain saturated carboxylic acids, short-chain dicarboxylic acids, and carboxylic acids with extra moieties such as 2-hydroxyisobutyric, crotonic, and lipoic acid residues).^6,8^ In line with this, HDACs were shown to catalyze a wider range of deacylation reactions in both protein and nonprotein substrates, such as polyamines.^6^

HDACs are divided into Zn^2+^-dependent deacylases consisting of classes I, II, and IV HDACs^9^ and nicotinamide adenine dinucleotide (NAD^+^)-dependent enzymes consisting of class III HDACs, also named sirtuins (SIRTs) due to their homology to the yeast silent information regulator 2 (Sir2).^10^ Some SIRT family members also possess broad-spectrum protein lysine deacylase, mono-ADP-ribosylase, and lipoamidase activities.^11^ Given their ability to catalyze the removal of many PTMs, sirtuins are involved in several biological processes, including DNA damage repair, aging, cell cycle regulation, gene expression, metabolism, longevity, and stress response.^12−15^ It is worth noting that epigenetic and metabolic pathways are tightly interconnected. Indeed, most enzymes that catalyze epigenetic modifications use crucial metabolites as cosubstrates (for example, *S*-adenosyl methionine, α-ketoglutarate, acetyl- and acyl-CoA, FAD/FADH_2_, and NAD^+^/NADH).^16^ Specifically, sirtuins require NAD^+^ as a cosubstrate for catalysis and are inhibited by NADH;^17^ as a result, they are sensitive to the intracellular NAD^+^/NADH ratio, thereby serving as sensors of cellular metabolic status. Under normal conditions, the NAD^+^/NADH ratio fluctuates modestly; nevertheless, it varies drastically under situations of nutrient deprivation, obesity, tumorigenesis, and aging. Consequently, changes in metabolism also influence gene expression and signaling pathways through the altered activity of sirtuins.

**Figure 1:**
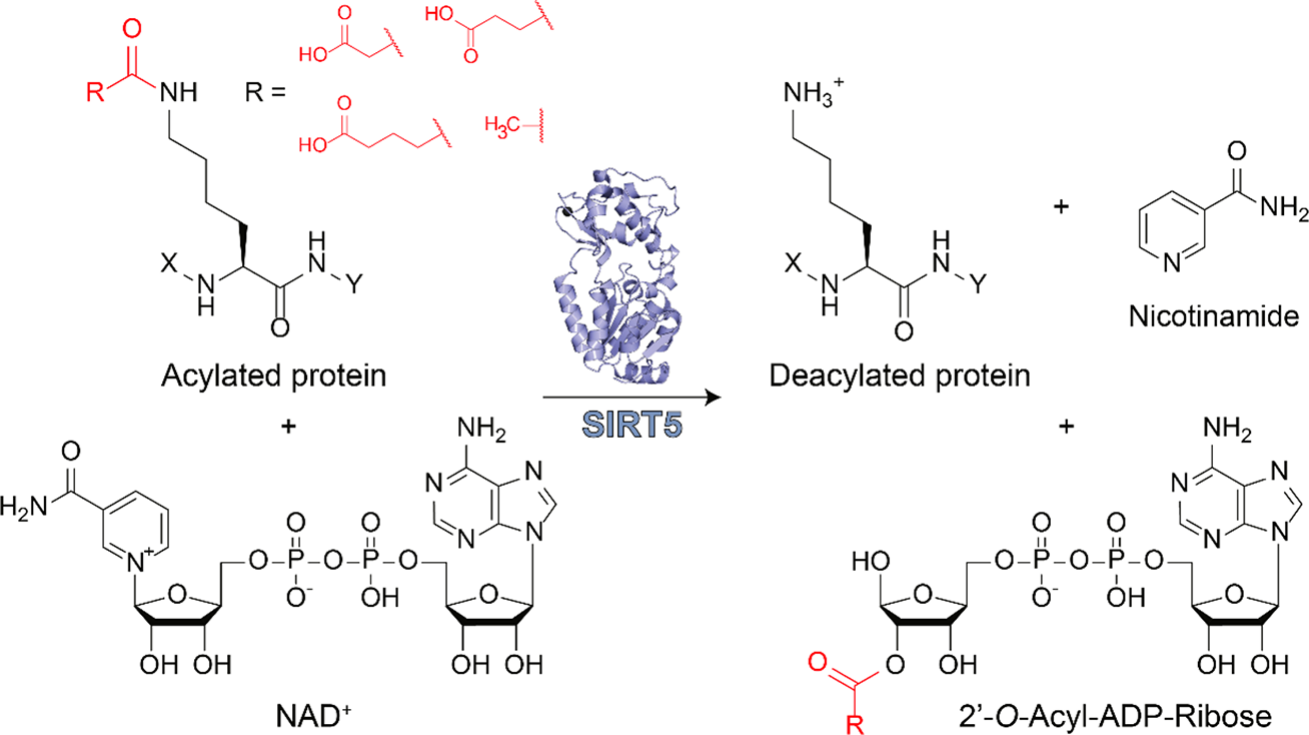
Deacylation reaction catalyzed by SIRT5. The acyl moiety is transferred to the NAD^+^ cosubstrate, yielding the corresponding 2′-*O*-acyl-ADP-ribose and nicotinamide.

In mammals, the sirtuin family includes seven isoforms (SIRT1–7)^18^ that possess highly conserved NAD^+^-binding and catalytic domains and differing *N*- and *C-*termini, which determine their substrate preference, enzymatic activity, and subcellular localization. SIRT1, SIRT6, and SIRT7 are mostly present in the nucleus, with SIRT7 being mainly a nucleolar protein. Among them, SIRT1 may also be found in the cytosol.^19−22^ SIRT2 is mainly cytoplasmic, although it may shuttle in the nucleus during mitosis, and an alternatively spliced isoform is constitutively present in the nucleus.^21,23,24^ Finally, SIRT3, SIRT4, and SIRT5 are predominantly found in the mitochondrial matrix.^19,25−28^ The sirtuin-mediated deacylation reaction employs NAD^+^ as a cosubstrate and produces, besides the deacylated product, 2′-*O*-acyl-ADP-ribose and nicotinamide, which can act as a physiological sirtuin inhibitor.^29^ Each sirtuin isoform exhibits a preference for different ε-*N*-acyl-lysine PTMs (Table 1). SIRT1–3 preferentially catalyze protein lysine deacetylation reactions.^19,30,31^ SIRT4 exhibits lipoamidase^32^ and mono-ADP-ribosyltransferase^27^ activities and has also the ability to cleave glutaryl, 3-methylglutaryl, 3-hydroxy-3-methyl-glutaryl (HMG), and 3-methylglutaconyl groups.^33,34^ SIRT6 exhibits a broad spectrum of deacylase activities and a mono-ADP-ribosyltransferase action,^35,36^ while SIRT7 exhibits deacetylation,^37−39^ desuccinylation,^40^ and deglutarylation^41^ activities. SIRT5 has been shown to selectively cleave negatively charged acyl lysine modifications, such as glutarate, succinate, and malonate, both *in vitro* and *in vivo*, although it also has weak deacetylase activity (Figure 1), with a catalytic efficiency roughly 1000-fold lower than those of the deacylation reactions.^18,42^ While lysine acetylation is catalyzed by many enzymes belonging to the HAT family,^43^ the identification of acyltransferases that catalyze the transfer of nonacetyl groups has remained elusive for many years. Recently, known enzymes that catalyze a diverse subset of reactions have been revealed to also exhibit lysine acyltransferase activity.^44,45^ These include carnitine palmitoyl transferase 1A (CPT1A) and HATs p300/CBP and general control nondepressible 5 (GCN5), which were shown to demonstrate succinyltransferase activity.^46−48^ GCN5 also exhibits lysine glutaryltransferase activity.^41^ In the case of long-chain fatty acids, the *N*-terminal glycine myristoyltransferases (NMTs) 1 and 2 were recently shown to also catalyze lysine myristoylation.^49^ Moreover, numerous lysine acyl modifications arise through a nonenzymatic mechanism involving the direct reaction of acyl-CoA species (especially 4- and 5-carbon negatively charged dicarboxyl CoA thioesters such as succinyl-CoA, glutaryl-CoA, methylglutaryl-CoA, and HMG-CoA) with lysine ε0amino groups under physiological conditions, particularly in the mitochondrial matrix.^50,51^ Finally, protein lipoylation, counteracted by SIRT4,^28^ is catalyzed by specific enzymes that either directly transfer lipoic acid to lysine ε0amino groups (LplA) or act indirectly via a stepwise mechanism whereby octanoic acid is transferred to lysine ε-amino groups by LipB or LplA, followed by the insertion of two sulfur atoms at C6 and C8 by LipA to form a complete lipoamide.^52^

**Table 1:** Summary of the Enzymatic Activity of Each SIRT Isoform

SIRT isoform	enzymatic activity
SIRT1	deacetylase
SIRT2	deacetylase
SIRT3	deacetylase
SIRT4	delipoylase, de-HMG-ase, deglutarylase, demethylglutarylase, demethylglutaconylase, mono-ADP-ribosylase
SIRT5	deglutarylase, desuccinylase, demalonylase, deacetylase
SIRT6	deacylase (long fatty acyl chains), deacetylase, mono-ADP-ribosylase
SIRT7	deacetylase, desuccinylase, deglutarylase

Several studies have indicated that SIRT5 participates in various biochemical pathways by regulating the activity of many metabolic enzymes such as carbamoylphosphate synthetase I (CPS1), which is important in ammonia detoxification,^53^ and 3-hydroxy-3-methylglutaryl-CoA synthetase 2 (HMGCS2), which is involved in the formation of ketone bodies.^54^ As a mitochondrial sirtuin, SIRT5 plays a pivotal role in mitochondrial metabolism, regulating amino acid degradation, cellular respiration,^55^ reactive oxygen species (ROS) management,^56^ fatty acid oxidation,^57,58^ and glycolysis.^59^ As a result, SIRT5 dysregulation can lead to a variety of diseases, such as metabolic (e.g., diabetes) and neurodegenerative disorders, cardiovascular pathologies, and cancer.^60−64^ Due to its wide-ranging functions, the modulation of SIRT5 activity has great potential for the treatment of these diseases. Consequently, SIRT5 is a valuable target for the development of modulators that, acting as either activators or inhibitors, may have significant therapeutic potential in various contexts. Here, we present an overview of SIRT5’s characteristics, structure, functional roles in both physiological and pathological cellular processes, and pharmacological modulation with the aim of suggesting new approaches for developing new potential SIRT5 modulators that may be used to treat SIRT5-related diseases.

## 2 Functional and Structural Features of SIRT5

SIRT5 is widely distributed in the human body and is mostly localized in the liver, heart, kidneys, brain, muscles, and testes.^19,53^ At the cellular level, SIRT5 is largely present in the mitochondrial matrix, although some studies have demonstrated its presence in the cytosol, nucleus, and peroxisomes.^39,57,65,66^ In line with this, high levels of several succinylated,^54,57,67^ glutarylated,^68^ and malonylated^59^ cytosolic and nuclear proteins were reported following SIRT5 deletion in mice, while the acetylation level was not affected.^11,68−71^ Interestingly, in humans, there are four different isoforms encoded by the *SIRT5* gene: SIRT5^iso1^, SIRT5^iso2^, and SIRT5^iso3^, which are localized in the mitochondria, and SIRT5^iso4^, which is localized in the cytosol. SIRT5^iso1^ is the most studied isoform, while SIRT5^iso2–4^ are rarely detected in human cells. Compared to SIRT5^iso1^, SIRT^iso2^ lacks 11 residues at the *C*-terminus, SIRT5^iso3^ lacks an internal sequence of 18 residues, and SIRT5^iso4^ lacks 108 *N*-terminal residues, including the mitochondrial localization tag.^66,72,73^

**Figure 2:**
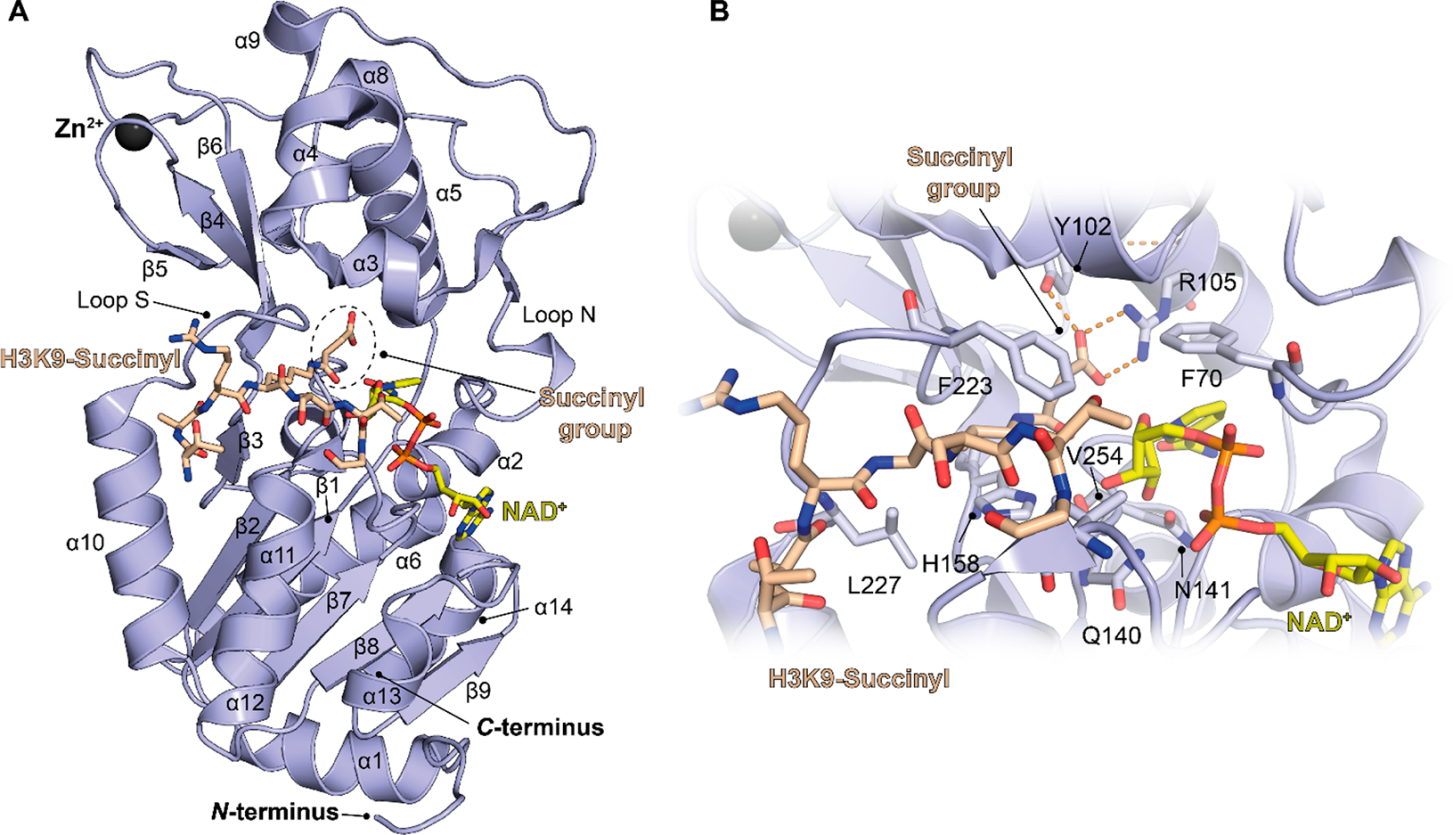
(A) Structure of SIRT5 in complex with the H3K9-succinyl peptide (beige) and bound NAD^+^ (yellow) (PDB ID 3RIY). (B) Focus on the catalytic pocket. The key interactions of the substrate peptide and NAD^+^ with SIRT5 residues are indicated. Dashed orange lines indicate polar interactions.

SIRT5 activity is controlled by two key metabolism regulators. The overexpression of peroxisome proliferator-activated receptor coactivator 1α (PGC-1α) leads to high levels of cellular SIRT5, whereas the activation of AMP-activated protein kinase (AMPK) causes SIRT5 downregulation.^74^ As previously stated, SIRT5 predominantly exhibits deglutarylase,^68^ desuccinylase,^54,57^ and demalonylase^59,70^ activities, but it also displays weak deacetylase activity toward different substrates (Figure 1).^42,69,75^ Specifically, using a CPS1-derived octapeptide appropriately modified at the lysine residues, Roessler and colleagues performed kinetic studies through a HPLC-based method to investigate the catalytic efficiencies of the various deacylation and deacetylation reactions. This analysis suggested that SIRT5 had the highest catalytic efficiency for deglutarylation (*k*_cat_/*K*_M_ = 18699 M^–1^ s^–1^), followed by desuccinylation (*k*_cat_/*K*_M_ = 13995 M^–1^ s^–1^) and demalonylation (*k*_cat_/*K*_M_ = 3758 M^–1^ s^–1^), while the deacetylation reaction was shown to be by far the least catalytically efficient (*k*_cat_/*K*_M_ = 16 M^–1^ s^–1^).^76^ Notably, adding a carboxylic group to the acyl chain did not produce massive changes in the apparent affinity for the SIRT5 catalytic site, since *K*_M_ remained in the same order of magnitude, but did increase the catalytic rate, as exemplified by the 50–200-fold increase in *k*_cat_. Given these results, it seems that the deacetylase activity of SIRT5 is negligible compared to the deacylase activity. Moreover, multiple studies indicate that deacylation, particularly desuccinylation, is the most relevant SIRT5-catalyzed reaction at the cellular level. However, some reports point toward the SIRT5-mediated deacetylation of certain substrates. Hence, the further characterization of SIRT5’s enzymatic activity at the cellular level would be necessary to understand whether SIRT5 genuinely has substrate-specific deacetylase activity or if these findings are due to SIRT5 overexpression or cross-reactivity with antiacetyllysine antibodies. To date, many crystal structures of SIRT5 in complex with substrates or small molecules have been released,^69,76−80^ thereby allowing the structural and functional characterization of the enzyme and aiding the design of specific modulators. By inspecting the crystal structure of SIRT5 in complex with the H3K9succ peptide and NAD^+^,^69^ we can observe that it consists of 14 α-helices and 9 β-strands that are organized to form a Rossmann fold and a Zn^2+^-binding domain. Between these two domains is a cleft that forms the catalytic region, which contains the binding sites of both the protein substrate and the cosubstrate NAD^+^. The Rossmann fold domain is comprised of six parallel β-strands that form a central β-sheet surrounded by nine α-helices. The Zn^2+^-binding domain contains five small α-helices and an antiparallel β-sheet formed by the β-strands (Figure 2A). The antiparallel β-sheet is stabilized by the presence of a Zn^2+^ ion coordinated with four Cys residues (Cys166, Cys169, Cys207, and Cys212).

The catalytic cleft is formed by several connecting loops between the Rossman fold and the Zn^2+^-binding domains. Loop S, which connects α10 of the Rossman fold domain with β6 of the Zn^2+^-binding domain, is crucial for substrate binding. Loop N, which connects α2 of the Rossman fold domain with α3 of the Zn^2+^-binding domain, is involved in NAD^+^ binding (Figure 2A). Many residues in this region are involved in substrate and cosubstrate binding. Among them, Phe223, Leu227, and Val254 define the hydrophobic entry gate for acyl-lysine, while Ala86, Tyr102, Arg105, and His158 directly interact with the acyl-lysine substrate. Gln140 and Asn141 interact with the ribose moiety of NAD^+^, whereas Asp143 binds the nicotinamide product (Figure 2B). In addition, the flexible residue Phe70 acts like a valve, facilitating NAD^+^ binding as well as nicotinamide release.^18,69^

Some of these structural features are conserved in SIRT1–3;^18,81−83^ for instance, the hydrophobic residues Phe223, Leu227, and Val254 are placed in the corresponding position in these orthologues. Conversely, SIRT5 possesses specific residues that characterize its substrate specificity and catalytic activity. In particular, the two nonhydrophobic residues Tyr102 and Arg105 localize deep into the substrate pocket, forming hydrogen bonds and electrostatic interactions with the negatively charged acyl-lysine substrate (Figure 2B). These residues precisely recognize glutaryl, succinyl, and malonyl groups, giving SIRT5 its specific deglutarylase, desuccinylase, and demalonylase activities, respectively.^69^ Another key residue for substrate recognition is Ala86, which is also specific to SIRT5 because SIRT1–3 bear a phenylalanine residue in the same position. The presence of alanine instead of phenylalanine makes the acyl-lysine binding pocket larger compared to those of other sirtuins, thereby making SIRT5 capable of binding bulkier acylated lysine substrates.^69,70^

## 3 Biological Activities and Disease Relevance of SIRT5

To date, it has been reported that SIRT5 regulates many processes involved in cellular metabolism and homeostasis. SIRT5 catalyzes NAD^+^-dependent deglutarylation, desuccinylation, and demalonylation of metabolic enzymes implicated in glycolysis;^59^ mitochondrial oxidative phosphorylation;^55^ fatty acid β-oxidation (FAO);^57,58^ ROS response;^56^ glutamine metabolism; and ammonia detoxification.^53,84,85^ In addition, SIRT5 expression is altered in a variety of cancer types, and it may behave as either a tumor promoter or a tumor suppressor. SIRT5 also plays significant roles in cardiac health maintenance and the neuronal stress response. A recent report suggested that SIRT5 is pivotal in facilitating the replication of severe acute respiratory syndrome coronavirus 2 (SARS-CoV-2), the etiologic agent causing the current COVID-19 pandemic.^86^ It is therefore apparent that SIRT5 has a rather pleiotropic nature, which is typical of other epigenetic proteins. Nonetheless, SIRT5 activity is mainly linked to the regulation of mitochondrial pathways. Hence, targeting SIRT5 would be especially useful in those settings where mitochondrial dysfunction is relevant. Moreover, the pleiotropic character of SIRT5 activities does not preclude SIRT5 from being considered a potential pharmacological target, since in certain contexts multiple SIRT50affected pathways concur to determine the same phenotype. In the next sections, we will provide detailed information on the molecular mechanisms connecting SIRT5’s activity and physiological and pathological roles and indicate the contexts where SIRT5 inhibition or activation may represent a viable therapeutic option.

### 3.1 Metabolism

**Figure 3:**
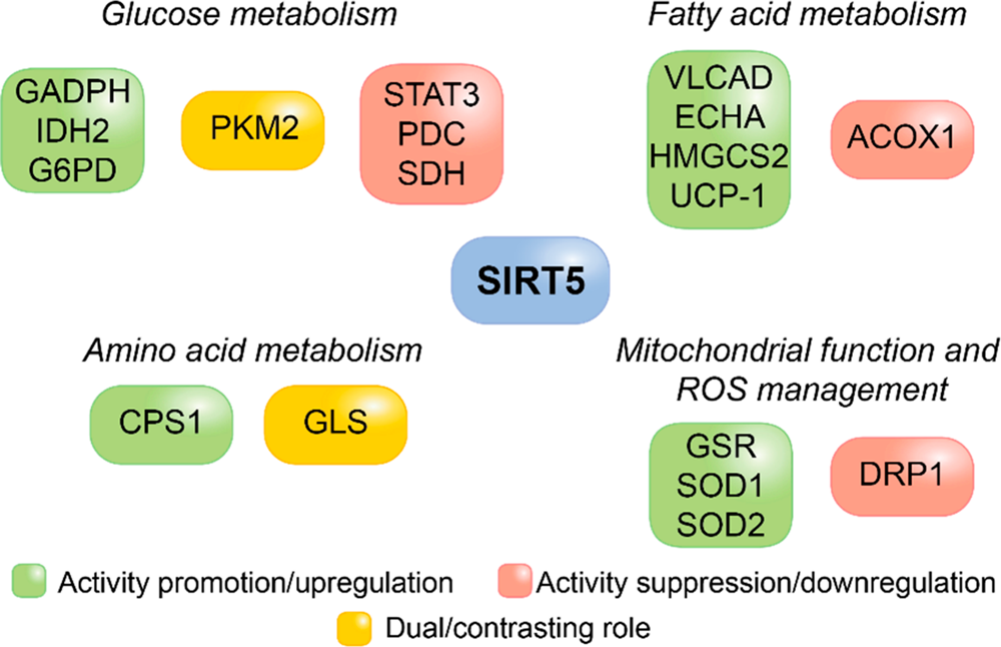
Involvement of proteins modulated by SIRT5 in the regulation of cellular metabolism, mitochondrial function, and the oxidative stress response.

As mentioned above, SIRT5 targets several proteins involved in glycolysis, gluconeogenesis, the tricarboxylic acid (TCA) cycle, and the electron transport chain (ETC), thus regulating many metabolic pathways. Notably, quantitative proteomic analyses showed that SIRT5 preferentially demalonylates glycolytic enzymes, including glyceraldehyde 3-phosphate dehydrogenase (GAPDH), thereby promoting glycolysis (Figure 3). In fact, Nishida et al. demonstrated that replacing Lys184 in GAPDH with glutamic acid, which mimics malonyl-lysine, leads to the inhibition of the enzymatic activity, thus suggesting that the enzyme works only after SIRT5-mediated demalonylation. Consistent with these findings, primary hepatocytes obtained from SIRT5 knockout (KO) mice displayed decreased glycolytic flux.^59^ These experiments indicated that SIRT5 regulates glucose metabolism. In addition, SIRT5 was also found to be involved in insulin sensitivity; indeed, high SIRT5 levels were found in adipose tissues and were linked with a high insulin response in monozygotic twins.^87^

Furthermore, SIRT5 has been shown to deacetylate the signal transducer and activator of transcription 3 (STAT3), suppressing its mitochondrial translocation and inhibiting its interaction with and activation of pyruvate dehydrogenase complex (PDC). This inhibits the catalytic activity of PDC, which consists of oxidizing pyruvate into acetyl-CoA, and subsequently prohibits acetyl-CoA from entering the TCA cycle.^88^ In the study, the authors also show that SIRT3 contributes, although to a much lesser extent, to STAT3 deacetylation. However, the biological significance of STAT3 deacetylation by SIRT3 was not further explored. In addition, the influence of SIRT5 on STAT3 deacylation (e.g., desuccinylation) was not assessed. Hence, given the weak deacetylase activity of SIRT5, we cannot exclude that it also acts as STAT3 desuccinylase. SIRT5 also inhibits PDC via direct desuccinylation (Figure 3), impairing pyruvate metabolism, causing a decrease in ATP production, and also resulting in the promotion of tumorigenesis. Consistent with this data, SIRT5 ablation resulted in increased ATP synthesis.^57^ However, SIRT5 loss in HEK293 cells is associated with a reduced pyruvate-dependent cellular respiration,^89^ thus suggesting that the role of SIRT5 in glucose metabolism is context-dependent.^90^

The double-faced role of SIRT5 in glycolysis has also been described in the regulation of pyruvate kinase M2 (PKM2), which transforms phosphoenolpyruvate into pyruvate. PKM2 exists in two different functional forms: as a tetramer it possesses strong pyruvate kinase activity, while as a dimer it is mainly localized in the nucleus, has weak pyruvate kinase activity, and mainly acting as a protein kinase.^91−94^ In a recent study, Wang and co-workers demonstrated that the SIRT5-mediated desuccinylation of PKM2 at Lys311 leads an augmented activity, thereby supporting the glycolytic flux.^94^ Conversely, Xiangyun and colleagues showed that SIRT5 desuccinylates PKM2 at Lys498 under oxidative stress conditions, inhibiting its activity (Figure 3), repressing glycolysis in lung cancer cells, and consequently readdressing the glucose flux into the pentose phosphate pathway.^95^ Another study reported that the desuccinylation of PKM2, under glucose deficiency conditions obstructs its translocation into mitochondria and facilitates the degradation of voltage-dependent anion channel 3 (VDAC3), thereby enhancing the opening of the mitochondrial permeability transition pore and finally leading to the apoptosis of colon cancer cells.^96^ In this case, the contrasting outcomes of these studies may depend on the different cell lines used or different types of induced stress conditions.

Another target of SIRT5 is the enzyme complex succinate dehydrogenase (SDH), also called respiratory complex II, which is involved in both the TCA cycle and the ETC. SDH catalyzes the oxidation of succinate to fumarate and simultaneously transforms ubiquinone to ubiquinol. SIRT5-mediated desuccinylation inhibits SDH activity (Figure 3) and consequently reduces succinate-dependent cellular respiration.^57^ Interestingly, Zhang and co-workers demonstrated that SIRT5 also desuccinylates various subunits of the ETC complexes and ATP-synthase after cardiolipin binding, thus promoting cellular respiration.^89^ Finally, SIRT5 has been shown to desuccinylate isocitrate dehydrogenase 2 (IDH2). This increases its activity for the oxidative decarboxylation of isocitrate to α-ketoglutarate in a NADP^+^-dependent manner, which produces NADPH and CO_2_ as byproducts.^97,98^

Concerning FAO, SIRT5 desuccinylates the very-long-chain acyl-CoA dehydrogenase (VLCAD) that catalyzes the initial step in the β-oxidation in mitochondria. Notably, SIRT5 cooperates with SIRT3, which deacetylates VLCAD at Lys299, stabilizing its localization and promoting the association of the cofactor flavin adenine dinucleotide (FAD).^58^ Overall, the two enzymes promote VLCAD activity (Figure 3) by facilitating the interaction with FAD and increasing its localization in the mitochondrial membrane. In line with this, reduced FAO was reported upon SIRT5 KO in mice.^54^ In addition, SIRT5 desuccinylates HMGCS2, thereby increasing its activity and stimulating ketone body formation under conditions of caloric restriction.^54^ SIRT5 supports another step of FAO by increasing the activity of enoyl-CoA hydratase (ECHA, Figure 3), which catalyzes the hydration of the double bond between C2 and C3 of enoyl-CoA.^67^

In mammals, there are two different types of adipose tissues: white adipose tissue (WAT) specializing in energy storage and release in the form of triglycerides and brown adipose tissue (BAT) containing multiple mitochondria devoted to the dissipation of energy through the expression of uncoupling protein 1 (UCP-1, Figure 3), which is involved in thermogenesis.^99,100^ Mitochondrial SIRT5 is largely expressed in BAT where it catalyzes protein demalonylation and desuccinylation, thus suggesting that it regulates BAT functions and thermogenesis.^101,102^ In mouse models, SIRT5 loss was found to reduce UCP-1 function, leading to protein hypersuccinylation and decreased levels of α-ketoglutarate and finally resulting in increased repressive histone methylation (H3K9me2 and H3K9me3) at the promoter region of *Prdm16*, a transcription factor that facilitates the expression of brown adipocyte genes.^103^ SIRT5 is also important in the differentiation of brown adipocytes and the conversion of white adipocytes to brown adipocytes.^103^ Overall, given the involvement of SIRT5 in BAT/WAT equilibrium and because BAT is a key regulator of glucose homeostasis, targeting SIRT5 may be a useful therapeutic approach against metabolic disorders such as obesity and type 2 diabetes.^64^

Various studies reported the key role of SIRT5 in the regulation of ammonia detoxification and amino acid catabolism through the deacylation and consequent activation of CPS1 (Figure 3).^53,68,69,104^ This enzyme catalyzes the conversion of ammonia into carbamoyl phosphate, the first reaction of the urea cycle.^105^ Under caloric restriction, SIRT5-overexpressing cells showed increased hepatic CPS1 activity due to high levels of SIRT5 mRNA in the liver.^104^ Conversely, SIRT5 KO mice exhibited lower CPS1 activities and enhanced ammonia levels in blood.^53,69^ SIRT5 also regulates ammonia production in nonliver cells, where it desuccinylates mitochondrial glutaminase (GLS); two studies have reported opposite outcomes (Figure 3). Polletta et al. demonstrated that the SIRT5-mediated desuccinylation of GLS inhibits its activity, thereby repressing the glutamine catabolism to glutamate and the generation of ammonia as a byproduct. The authors proposed Lys245 and Lys320 as possible succinylation sites that may be accessible to the SIRT5 catalytic pocket. Since it was reported that ammonia could induce autophagy and mitophagy in tumor cells, the SIRT5-mediated inhibition of GLS could overcome this protective mechanism for tumor cells, suggesting a tumor suppressor role for SIRT5 in this context.^84^ Conversely, another study suggested that SIRT5-mediated desuccinylation at Lys164 protects GLS from ubiquitination at Lys164 and the consequent proteasomal degradation, thereby stabilizing it and supporting glutamine catabolism.^85^

### 3.2 Mitochondrial Function and Oxidative Stress

The fact that SIRT5’s deacylating activity is reliant on NAD^+^, a major redox signaling molecule, supports the idea that it is a key player in the regulation of cellular redox homeostasis. Indeed, since NAD^+^ is a key electron acceptor in multiple enzymatic reactions, the NAD^+^/NADH ratio is a crucial factor for redox pathways and, therefore, the regulation of ROS levels.

Guedouari et al. reported that SIRT5 regulates many mitochondrial processes, such as elongation, fusion, and division. Indeed, SIRT5-depleted mouse embryonic fibroblasts (MEFs) displayed augmented mitochondrial fragmentation and mitophagy under starvation conditions, along with an increase of dynamin-related protein 1 (DRP1) levels (Figure 3). This indicates that SIRT5 defends mitochondria from starvation-induced autophagy and degradation.^106^

SIRT5 has a significant role in reducing ROS levels through modulating different enzymes. These include the previously mentioned glycolytic enzymes and glucose-6-phosphate dehydrogenase (G6PD), which converts glucose 6-phosphate to ribose 5-phosphate for the biosynthesis of nucleotides in the pentose phosphate pathway. They both produce NADPH as a byproduct, which is important for the reduction of oxidized glutathione (GSSG) to reduced glutathione (GSH). GSH in turn reduces cellular ROS levels. SIRT5 desuccinylates and deglutarylates IDH2 and G6PD, respectively, activating these enzymes (Figure 3) and promoting NADPH production.^97^ In line with this, SIRT5 KO or knockdown leads to significantly decreased NADPH and GSH levels, leading to an impairment of the ROS scavenging capability and increased cell vulnerability to oxidative stress.^97^ Furthermore, SIRT5 deficiency was shown to be correlated with lower levels of glutathione reductase (GSR),^62^ the enzyme that converts GSSG to GSH.^107^ In particular, in nonsmall cell lung cancer (NSCLC) cells, SIRT5 knockdown resulted in reduced GSR expression.^62^

SIRT5 attenuates oxidative stress by targeting peroxisomal acyl-CoA oxidase 1 (ACOX1), a key enzyme involved in FAO that contributes to H_2_O_2_ production.^108^ ACOX1 is functional as a dimer, and its dimerization is inhibited by SIRT5 desuccinylation, thereby blocking H_2_O_2_ production and mitigating oxidative stress.^39^ SIRT5 was also reported to regulate oxidative stress via the deacetylation of the Forkhead protein FOXO3a, thus promoting its shuttling into the nucleus and facilitating the expression of antioxidant defense-related genes.^109^ However, it should be noticed that FOXO3a is also deacetylated by SIRT1–3, which possess higher deacetylase activities than SIRT5. Moreover, SIRT5-mediated desuccinylation activates Cu/Zn superoxide dismutase 1 (SOD1, Figure 3), and there is a consequent increase in ROS detoxification.^56^ Overall, these findings suggest that SIRT5 has a pivotal role in regulating cellular mechanisms to protect cells from oxidative stress.

### 3.3 Neurodegeneration

Mitochondrial functions such as energy production, apoptotic signaling, redox homeostasis, and oxidative phosphorylation are crucial for neuronal health. Consequently, the dysfunction of these processes is connected with the onset of many neurodegenerative diseases, including Parkinson’s disease (PD), Alzheimer’s disease (AD), and epileptic disorders.^110^ In this context, SIRT5 plays neuroprotective roles, as exemplified by several studies.

Following exposure to kainate, a glutamate analogue that exters neuroexcitatory and epileptogenic effects,^111^ SIRT5 expression increased in the hippocampus, thereby ensuring neuroprotection against the formation of astrogliosis. Consistent with this data, the depletion of SIRT5 in kainate-exposed mice leads to hippocampal neuronal loss and a severe response to epileptic seizure, which is caused by kainate activity on glutamate receptors.^40^ Interestingly, the protective role of SIRT5 in this context seems unrelated to its function in ROS detoxification.

1-Methyl-4-phenyl-1,2,3,6-tetrahydropyridine (MPTP) is chemical tool widely employed to induce PD symptoms in animal models. It is a prodrug of the neurotoxin 1-methyl-4-phenylpyridinium (MPP^+^), which causes the degeneration of dopaminergic neurons in the *substantia nigra* by increasing ROS levels and inducing cell death.^112,113^ Notably, treatment with MPTP induced SIRT5 expression in the brain of treated mice. Conversely, a SIRT5 deficiency in mouse brain striata exacerbated the MPTP-induced loss of nigrostriatal dopaminergic neurons. This was associated with the reduced expression of the mitochondrial antioxidant enzyme manganese superoxide dismutase 2 (SOD2, Figure 3).^61^ These results suggest that SIRT5 activity contributes to ROS scavenging in nigrostriatal dopaminergic neurons and alleviates the effects of MPTP.

Finally, SIRT5 seems to have a protective role also in the context of AD. Indeed, AD mouse models displayed the downregulation of SIRT5 and impaired autophagy, which was reversed by SIRT5 overexpression.^114^ In addition, SIRT5 expression was associated with elevated SOD activity, lower ROS levels, and diminished apoptosis both *in vitro* and *in vivo*. Neuron damage and inflammation were also lower in AD brains that expressed higher SIRT5 levels, which may be a consequence of the inhibition of astrocytes and microglia activation. Overall, these results indicate that SIRT5 activity mitigates neuron damage by suppressing oxidative stress and decreasing the activity of astrocytes and microglia.

### 3.4 Cardiovascular Regulation

We previously mentioned that the deficiency of SIRT5 in cardiac tissue results in increased levels of succinylated lysine proteins^67,115^ including SDH,^57^ which is inhibited by SIRT5-mediated desuccinylation (Figure 3). Interestingly, SIRT5 deficiency has been associated with an increased predisposition to myocardial ischemia-reperfusion injury.^116^ In addition, treatment with dimethyl malonate, a precursor of the SDH inhibitor malonate, led to reduced superoxide production in SIRT5 KO hearts, confirming the key role of SIRT5 in regulating ROS levels even at the cardiac level.^116^ In line with this, another study with both *in vitro* and *in vivo* models confirmed that the inhibition of SDH in the heart is protective against cardiac myocardial ischemia-reperfusion damage.^117^

Furthermore, SIRT5 has a protective role for cardiomyocytes, since it suppresses oxidative-stress-induced apoptosis through its interaction with the antiapoptotic factor Bcl-XL.^118^ SIRT5 also plays a significant role in the cardiac stress response. In a model of hypertrophy induced by pressure overload as a consequence of traverse aortic constriction, SIRT5 loss was associated with a twofold increase in succinylation in more than 750 proteins, along with cardiac dysfunction and higher mortality rates.^115^

As mentioned above, SIRT5 activates ECHA, an enzyme crucial for myocardial fatty acid metabolism, through desuccinylation (Figure 3). Hence, SIRT5 ablation impairs cardiac FAO and reduces ATP production in conditions where energy is particularly needed, such as during physical exercise or fasting conditions. In addition, SIRT5 KO causes cardiac hypertrophy and an altered echocardiogram profile.^67^ Overall, these results suggest the importance of SIRT5 activity in cardiac tissue, since its deletion or downregulation may impair heart functionality.

### 3.5 COVID-19

Recently, SIRT5 was shown to interact with the nonstructural protein 14 (Nsp14) from SARS-CoV-2, a highly conserved enzyme required for viral replication.^86^ Nsp14 interacts with Nsp10, which stabilizes its *N*-terminal domain possessing 3′–5′-exoribonuclease activity. The Nsp14–Nsp10 complex is therefore essential for exoribonuclease activity. Nsp14 also possesses a C-terminal domain that displays RNA cap guanine *N*7-methyltransferase activity, which is not influenced by Nsp10 binding. SIRT5 was shown to interact with Nsp14 but not Nsp10, suggesting the formation of an alternative complex. Notably, SIRT5 catalytic activity is necessary for this interaction, as suggested by mutation experiments or pharmacological inhibition (see compound **3d** in section 4.1). However, Nsp14 does not seem to be a direct substrate of SIRT5, and a clear molecular function of the Nsp14–SIRT5 complex could not be revealed. Nonetheless, at the cellular level, SIRT5 KO or pharmacological inhibition reduced SARS-CoV-2 levels. In addition, SIRT5 KO led to higher levels of immunity and a better antiviral response, indicating that SIRT5 also has a role in SARS-CoV-2 infection that goes beyond its interaction with Nsp14. Overall, this study uncovered an unusual type of interaction and points toward the key role of SIRT5 in viral replication, suggesting that SIRT5 inhibition could be a useful strategy to combat COVID-19, most likely in combination with other therapeutics.^86^

### 3.6 Double-Faced Role in Cancer

Like other human sirtuins, SIRT5 is involved in different processes, including the maintenance of genomic stability, metabolism, and tumor microenvironment regulation.^119,120^ Hence, it is not surprising that SIRT5 may have a tumor-promoting or tumor-suppressing role depending on the context and cancer type.

In the next sections, we report different cases in which SIRT5 exhibits either a tumor-suppressor or tumor-promoter function. Notably, in lung cancer,^56,62,88,121^ hepatocellular carcinoma (HCC),^39,122−124^ and breast cancer,^84,85^ SIRT5 displays a dichotomous role, further indicating that its activity is dependent strictly on the specific context and not only the type of tissue or cancer.

#### 3.6.1 Tumor Suppressor Role of SIRT5 in Cancer

As demonstrated by *in vitro* and *in vivo* experiments, SIRT5 exerts tumor suppressor functions in glioma, where its desuccinylase activity plays pivotal roles in maintaining mitochondrial functions and arresting cell proliferation.^55^

**Figure 4:**
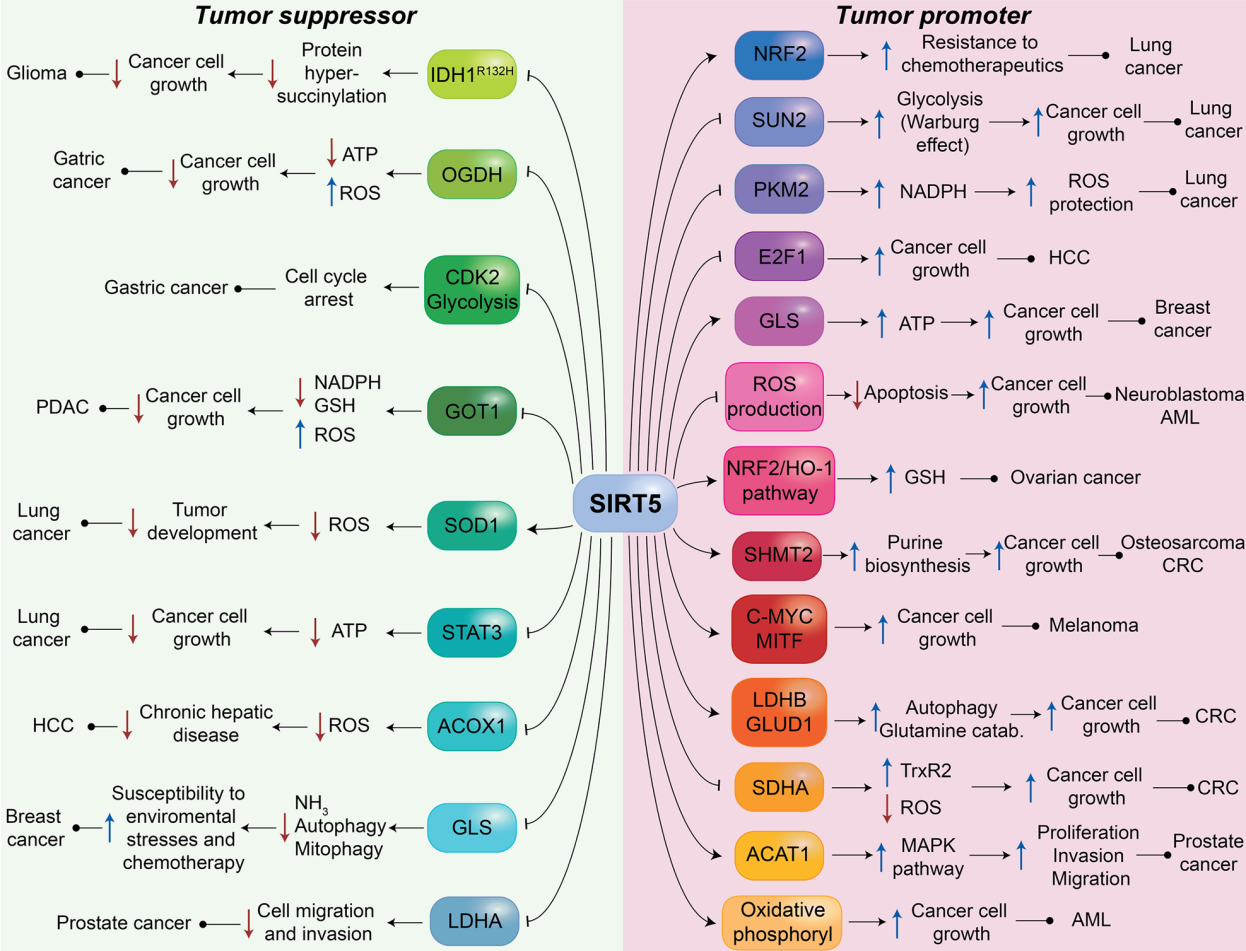
Roles of SIRT5 in cancer. The figure depicts the main proteins and pathways regulated by SIRT5, which exerts both tumor-suppressing and tumor-promoting functions. Several mechanisms are implicated, including the regulation of glycolysis, FAO, amino acid metabolism, ATP production, ROS detoxification, apoptosis, and autophagy.

Clark and colleagues reported the presence of mutant *IDH1* and *IDH2* in different types of cancer such as acute myeloid leukemia (AML), chondrosarcoma, and glioma.^125^ Instead of catalyzing the conversion of isocitrate to α-ketoglutarate, mutant IDH1 and IDH2 convert α-ketoglutarate to *R*-2-hydroxyglutarate.^98,126^ This derivative is proposed to promote cancer progression and protect tumor cells from apoptosis by inhibiting α-ketoglutarate-dependent dioxygenases^127^ and SDH, leading to an upsurge of succinyl-CoA levels and the consequent aberrant succinylation of mitochondrial proteins.^55^ Furthermore, in glioma cells presenting the R132H mutation of IDH1, protein hypersuccinylation leads to the accumulation of Bcl-2, which promotes apoptotic resistance.^55^ Conversely, SIRT5 overexpression in glioma cells decreased protein succinylation and reduced cell growth both *in vitro* and *in vivo* (Figure 4).^55^

In gastric cancer, SIRT5 overexpression inhibits oxoglutarate dehydrogenase (OGDH), thus decreasing ATP production, increasing ROS levels, and leading to the inhibition of cancer cell proliferation and migration.^128^ Furthermore, enhanced SIRT5 activity leads to cell cycle arrest at the G1/S phase in tumor cells due to the negative modulation of cyclin-dependent kinase 2 (CDK2) and the inhibition of glycolysis (Figure 4).^129^

A recent report by Hu et al. reported that SIRT5 acts as a tumor suppressor in pancreatic ductal adenocarcinoma (PDAC).^130^ PDAC cells with *KRAS* mutations metabolize glutamine following the GOT2/GOT1/ME1 pathway, a dispensable pathway for the other cells. It was reported that SIRT5 deacetylates aspartate aminotransferase GOT1, predominantly at Lys369, thus inhibiting its activity and decreasing the relative abundance of glutamine or glutathione metabolism intermediates. GOT1 catalyzes the conversion of α-ketoglutarate and aspartate into glutamate and oxaloacetate in the cytosol, increasing NADPH and GSH production to maintain redox homeostasis and facilitate PDAC cell growth (Figure 4). Accordingly, SIRT5 loss leads to a reduction in ROS levels and the consequent proliferation of tumor cells. Notably, it was found that SIRT5 expression is downregulated in both human PDAC tissues and murine pancreatic tumors and is associated with cancer progression and poor prognosis. Furthermore, SIRT5 KO mice expressing *KRAS* or *KRAS/p53* oncogenic mutations exhibited an acceleration in tumor onset and significantly enhanced cancer cell proliferation in a caerulein-induced pancreatitis model in the absence of caerulein. These findings show that SIRT5 may be a tumor suppressor in this type of cancer and that its pharmacological activation (see compound **14**, section 4.1) impairs GOT1 activity and reduces PDAC cell viability.^130^ Hence, activating SIRT5 could be a promising strategy to target PDAC.

As mentioned in the previous section, SIRT5 desuccinylates and activates SOD1, thereby exerting a key function in ROS detoxification. Lin et al. observed that SOD1 succinylation increased lung cancer cell proliferation (Figure 4). In line with this, cells expressing a SOD1 mutant resistant to succinylation showed decreased growth rates, suggesting the protective role of SIRT5 in this setting.^56^ In addition, in lung cancer A549 cells, SIRT5 is downregulated, resulting in the acetylation and mitochondrial translocation of STAT3. This accelerates the transformation of pyruvate to acetyl-CoA through the interaction with PDC, thus promoting ATP production that sustains cell growth.^88^

We previously stated that SIRT5 desuccinylates and inhibits the peroxisomal enzyme ACOX1,^39^ thus reducing the production of H_2_O_2_ and consequently alleviating cellular oxidative stress.^108^ The excessive activation of ACOX1 leads to oxidative DNA damage and alters FAO and redox homeostasis, which causes chronic hepatic disease and finally leads to the insurgence of HCC (Figure 4).^39^ Another study also indicated that SIRT5 expression is lower in primary liver cancer tissue compared to normal hepatic tissues.^122^ This causes intensified succinylation and the consequent activation of ACOX1, finally promoting HCC progression due to elevated H_2_O_2_ production and oxidative stress (Figure 4).^39^ Hence, these studies suggest that SIRT5 activity may prevent the development of HCC.

As previously mentioned, SIRT5 is involved in ammonia detoxification through desuccinylation and the consequent inhibition of GLS, which catalyzes the hydrolysis of glutamine to glutamate and produces ammonia as a byproduct.^84^ Notably, breast cancer cells MDA-MB-231 and C2C12 overexpressing SIRT5 were characterized by decreased ammonia levels, with a consequent reduction of ammonia-induced autophagy and mitophagy (Figure 4). These mechanisms play a defensive role against chemotherapy or stress mechanisms such as hypoxia or fasting.^84^ Importantly, in cancer cells, glutamine catabolism is necessary for ATP production and lipid biosynthesis to support cell proliferation. Indeed, glutamine is crucial for the anaplerotic replenishment of the TCA cycle through its catabolic product α-ketoglutarate.^131^ Hence, in these cases, SIRT5 acts as a tumor suppressor, rendering tumor cells more susceptible to chemotherapeutics and environmental stresses and causing a decrease in ATP production.

SIRT5 was found to be downregulated in androgen-independent prostate cancer cells (PC-3 and PC-3M), with its expression being lower in more advanced cancers. Furthermore, inhibiting SIRT5 with a peptide-based inhibitor (compound **3d**, section 4.1) increased PC-3 cell migration and invasion, thereby confirming its tumor suppressive role in this context. In line with this, SIRT5 KOincreases PC-3 cell proliferation, migration, and invasion. The observed effects were ascribed to the higher activity of lactate dehydrogenase (LDH) A, which is activated upon succinylation at Lys118 and is a demonstrated substrate of SIRT5. Nonetheless, no mechanistic insight was provided regarding the role of LDHA in the onset and progression of prostate cancer.^132^

#### 3.6.2 Tumor-Promoting Role of SIRT5 in Cancer

SIRT5 may also play a tumor-promoting function in lung cancer via different mechanisms. Indeed, a recent study indicated that SIRT5 is overexpressed in NSCLC cells, which is associated with poor prognosis. Consistent with this, SIRT5 downregulation suppressed tumor cell growth and differentiation^121^ and sensitized lung cancer cells to genotoxic drugs such as cisplatin, 5-fluorouracil, and bleomycin both *in vitro* and *in vivo*.^62^ Moreover, SIRT5 ablation decreased the expression of NRF2 (Figure 4), a transcription factor involved in the regulation of genes that defend cells from oxidative stress and xenobiotics, including drug resistance genes.^62^

SIRT5 negatively regulates the expression of SAD1/UNC84 domain protein 2 (SUN2) (Figure 4), an important component of the linker of the nucleoskeleton and cytoskeleton (LINC) complex.^121^ SUN2 inhibits the Warburg effect, a metabolic alteration in which ATP is produced mainly from glycolysis rather than oxidative phosphorylation, thereby generating immediate energy to support cancer cell proliferation.^133^ SUN2 activity facilitates the suppression of cancer cell growth, metastasis, and the increased susceptibility to apoptosis induced by cisplatin. Overall, in this context, SIRT5 seems to play an oncogenic role by impairing SUN2 activity and sustaining tumor growth via the Warburg effect.^121^

SIRT5 may also play a tumor-promoting role in lung cancer via inhibiting PKM2 (Figure 4). The impairment of PKM2 activity results in a diminished glycolytic flux in tumor cells but consequently promotes the pentose phosphate pathway, yielding higher NADPH levels. This thus protects cancer cells from oxidative stress and facilitates their proliferation. Moreover, PKM2 hypersuccinylation led to the repression of tumor development, consistent with the fact that SIRT5 ablation or cell treatment with the nonselective sirtuin inhibitor suramin (compound **7**, section 4.1) induced PKM2 activity and thus suppressed the proliferation of A549 lung cancer cells.^95^ Similarly, SIRT5 KO in HCC is correlated with enhanced apoptosis and reduced cell proliferation and invasion, while its overexpression is associated with poor prognosis.^124^ SIRT5 was shown to negatively regulate the expression of E2F1 (Figure 4), an oncosuppressor involved in cell cycle regulation,^134^ thereby suggesting that HCC tumor progression could be supported by SIRT5 via the downregulation of E2F1.^124^

Different from what was described previously about SIRT5’s role in breast cancer, Greene et al. suggested that SIRT5 plays an oncogenic role through stabilizing GLS against ubiquitination and proteasomal degradation (Figure 4).^85^ This in turn supports glutamine catabolism and the consequent obtainment of α-ketoglutarate, which enters the TCA cycle that leads to ATP production. SIRT5 was shown to be upregulated during the cancerous transformation and promoted tumorigenesis and cell proliferation. In addition, increased SIRT5 expression in human breast tumors was correlated with poor prognosis for patients.^85^ Consistent with this, the pharmacological inhibition of SIRT5 strongly impaired the cell proliferation and anchorage-independent growth of MCF7 and MDA-MB-231 breast cancer cells (see compounds **2a** and **2b** in section 4.1).^135^

Liang et al. reported that SIRT5 was overexpressed in cultured SH-EP neuroblastoma cells, where it counteracted oxidative stress by reducing ROS levels and preventing apoptosis (Figure 4), thus exerting a tumor-promoting function.^136^ SIRT5 is also overexpressed in ovarian cancer,^137^ where it protects tumor cells from genotoxic drugs such as cisplatin by modulating the NRF2/HO-1 pathway, which in turn increases the cellular levels of the ROS scavenger GSH (Figure 4).^138^

Yang et al. showed that SIRT5-mediated desuccinylation at Lys280 activates the catabolizing enzyme serine hydroxymethyltransferase 2 (SHMT2), which in turn promotes tumor progression in osteosarcoma U2OS and colorectal carcinoma (CRC) HCT116 cells (Figure 4).^139^ Indeed, SIRT5 KO or the expression of the succinylation mimetic SHMT2 mutant (K280E) resulted in the suppression of tumor growth both *in vitro* and *in vivo*.^139^ SHMT2 is a crucial enzyme involved in one-carbon-unit metabolism that catalyzes the conversion of serine into glycine using tetrahydrofolate (THF) as a cosubstrate, which is converted to *N*^5^,*N*^10^-methylene-THF, a key intermediate of purine biosynthesis.^140^ Similarly, SIRT5 was found to activate the one-carbon-unit metabolism in melanoma. The activation of this pathway, along with the promotion of the expression of pro-survival genes such as *c-MYC* and *MITF*, was shown to sustain melanoma cell growth (Figure 4).^141^ In particular, SIRT5 was shown to promote the proliferation and survival of different cutaneous melanoma cell lines and a uveal melanoma cell line, a subtype that develops in the eye. In addition, SIRT5 was essential for tumor development in both melanoma mouse xenografts and the autochthonous *BRAF PTEN*-driven melanoma mouse model. SIRT5 was also found to regulate both the methylation and acetylation of histone, which in turn facilitate the expression of the above-mentioned *c-MYC* and *MITF*, respectively.^141^

In another study, high SIRT5 expression in CRC cells was associated with increased autophagy, which promotes tumor onset and progression.^142^ Mechanistically, SIRT5 deacetylates and activates LDHB, which promotes the conversion of lactate and NAD^+^ to pyruvate, NADH, and H^+^. The generated protons promote lysosomal acidification and consequent autophagy (Figure 4). Consistent with this, SIRT5 KO or treatment with the nonselective SIRT5 inhibitor GW5074 (see compound **11** in section 4.1) augmented LDHB acetylation at Lys329 and inhibited LDHB activity, which reduced autophagy and CRC cell growth both *in vitro* and *in vivo*. It should be noted that while the effects of SIRT5 KO are clearly related to the loss of SIRT5 activity, the consequences of GW5074 treatment cannot be unambiguously connected to SIRT5 inhibition or downregulation given the lack of selectivity of the compound. Furthermore, SIRT5-mediated deglutarylation and the consequent activation of glutamate dehydrogenase 1 (GLUD1) stimulate glutamine catabolism, supporting CRC proliferation (Figure 4).^143^ In line with this, SIRT5 knockdown in HCT116 and LoVo CRC cell lines led to the inhibition of cell proliferation.^143^ In addition, CRC cells expressing both SIRT5 and wild-type KRAS display resistance to anticancer agents like cetuximab. High SIRT5 expression in CRC patients expressing wild-type KRAS is also associated with increased tumor recurrence and poor survival.^144^ In this context, it was shown that drug resistance was gained by the activation of the ROS scavenger protein thioredoxin reductase 2 (TrxR2).^144^ Mechanistically, the SIRT5-mediated desuccinylation of succinate dehydrogenase complex subunit A (SDHA) and the inhibition of its enzymatic activity lead to higher levels of succinate, which determines TrxR2 activation (Figure 4). Through this mechanism, SIRT5 protects tumor cells from oxidative damage and promotes their proliferation.^144^ In line with this, SIRT5 silencing leads to the activation and hypersuccinylation of SDHA, thereby suppressing clear cell renal cell carcinoma proliferation.^145^

Different from what previously reported, SIRT5 was shown to possess tumor-promoter activity in prostate cancer, where it activates acetyl-CoA acetyltransferase 1 (ACAT1) and thus stimulates the mitogen-activated protein kinase (MAPK) pathway, leading to enhanced proliferation, invasion, and migration.^146^ SIRT5 also has a critical role in the development of AML, where its activity promotes cancer cell survival by reducing oxidative stress and sustaining oxidative phosphorylation and glutamine catabolism.^147^ In line with this, SIRT5 knockdown decreases colony formation and enhances apoptosis in a wide range of AML cell lines, and the pharmacological inhibition of SIRT5 impairs cell proliferation and induces apoptosis in SIRT5-dependent AML cells such as OCI-AML2, SKM-1, and MOLM-13 (see compounds **3b**, **3d**, and **3i**, respectively, in section 4.1).^147,148^ Similarly, SIRT5 expression is necessary for tumor insurgence and growth in both xenograft and syngeneic AML mouse models.^147^ Finally, a recent study also revealed that the tumor suppressor p53 is succinylated at Lys120;^149^ this residue was also previously identified as an acetylation site of KAT8, Tip60, and NAT10.^3,43^ In this case, SIRT5 mediates p53 desuccinylation, which results in its inhibition and the consequent suppression of both the expression of p53 target genes and p53-induced apoptosis. These data suggest that SIRT5 may also act as a tumor promoter by suppressing the functions of p53.^149^

## 4 Pharmacological Modulation of SIRT5

Given the involvement of SIRT5 as a regulator of different pathways, many research groups have investigated the possibility of targeting SIRT5 via inhibitors or activators. So far, research has been mainly focused on SIRT5 inhibitors used as either chemical tools to phenocopy SIRT5 knockdown or lead molecules for the development of novel potential therapeutics. On the other hand, a recent study described the first SIRT5 activator that has been used in the context of cancer, specifically PDAC, where SIRT5 plays an oncosuppressor role.^130^ This indicates that there is increased interest in developing both inhibitors and activators, thereby enabling a better understanding of SIRT5’s function and paving the way to personalized approaches. In the next section, we will initially examine the most relevant SIRT5 inhibitors and then discuss a recently reported activator.

### 4.1 SIRT5 Inhibitors

#### 4.1.1 Peptide and Amino Acid Inhibitors

**Figure 5:**
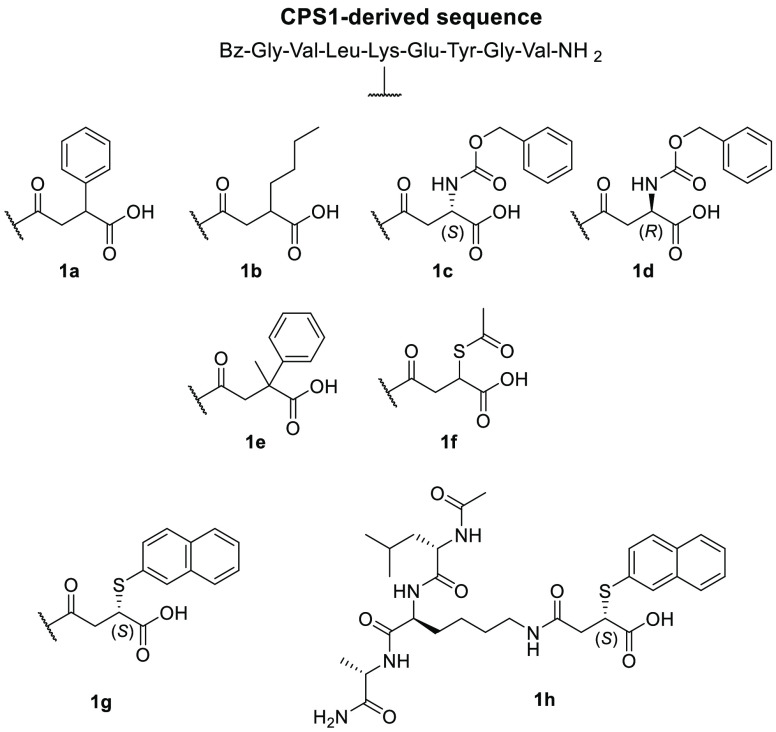
Structures of CPS1-derived peptidic SIRT5 inhibitors.

Starting from the analysis of the SIRT5 crystal structure that provided important information about its catalytic site, Roessler et al. synthesized various peptide-based analogues based on a CPS1-derived sequence (Figure 5) that served as a SIRT5 substrate in its acetylated form.^76^ All these compounds possess a succinyl residue at the lysine side chain, which was shown to interact with Tyr102 and Arg105 in the catalytic site. This dicarboxylic acyl portion gives the compounds the optimal chain length to allow the carboxyl group to form a salt bridge with Arg105 as well as a hydrogen bond to Tyr102. To obtain compounds that could impair NAD^+^ binding in the so-called C-pocket, which accommodates nicotinamide, the authors introduced bulky moieties in the C3 position of the succinyl chain. This approach initially led to compound **1a**, which had a phenyl ring on C3 and possessed a *K*_D_ value of 8.20 μM and *K*_*i*_ value of 100 μM for lysine desuccinylation. Compound **1b**, bearing a *n*-butyl chain on succinyl C3, displayed a great increase in inhibitory potency (*K*_*i*_ = 17.2 μM). Both peptides were crystallized in complex with zebrafish SIRT5 (zSIRT5) and showed similar binding modes, with the substituent at C3 pointing toward the binding site. In an attempt to move the phenyl moiety further inside the C-pocket, the authors inserted a methylcarbamate linker between the phenyl and succinyl groups, yielding compounds **1c** and **1d** with *S-* and *R-* configurations on C3, respectively. Compound **1c** displayed a *K*_D_ value of 5.78 μM that was associated with a *K*_*i*_ value for desuccinylation of 38.1 μM, almost threefold lower compared to that of **1a**. Although no thermodynamic constants were provided for compound **1d**, it was shown to possess a reduced affinity for SIRT5, thereby suggesting that the *R*-configuration is not optimal for the interaction with the C-pocket. The cocrystal structure of zSIRT5 in complex with **1d** showed that extending the linker moved the phenyl ring deeper into the C-pocket, thereby mimicking the nicotinamide binding. Hence, the augmented potency may be ascribed to both interactions with the key residues in the catalytic site and the steric hindrance that blocks the NAD^+^ binding. Compound **1e**, consisting of a derivative of **1a** bearing an additional methyl group on C3, displayed an almost 25-fold increase in inhibitory potency, with a *K*_*i*_ value of 4.3 μM (desuccinylation). Tested at a concentration of 50 μM against SIRT1–3, **1e** displayed less than 1% inhibition for SIRT1 and SIRT3 and ∼4% inhibition for SIRT2, showing great selectivity for SIRT5 (Table 2). Another active compound, although less potent than **1e**, is **1f**, which has a thioacetic residue on succinyl C3 and displays a *K*_*i*_ value of 10.6 μM (desuccinylation).^76^

**Table 2:**
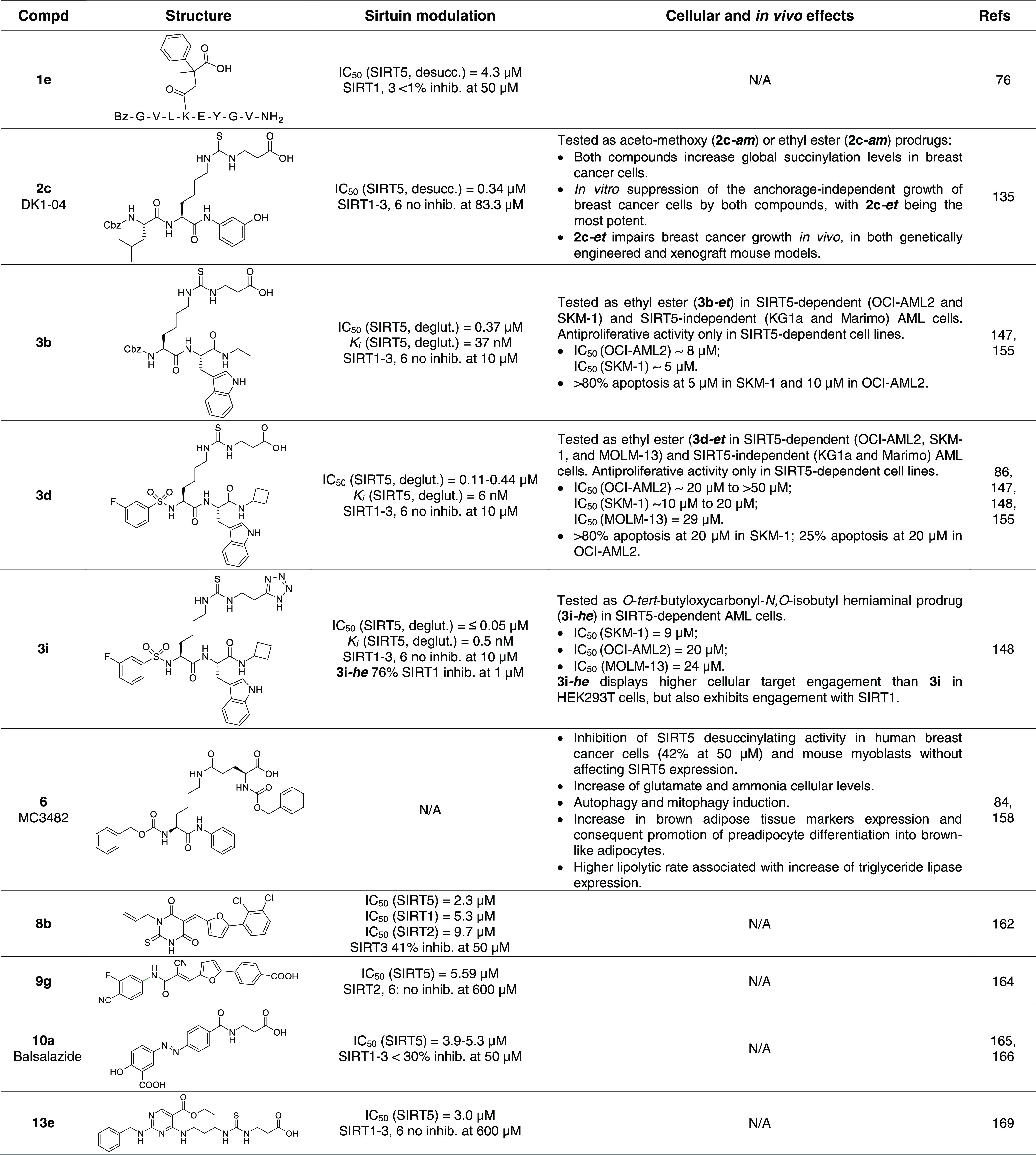
Most Relevant SIRT5 Inhibitors

In another study, the same research team analyzed the 3-(arylthio)succinyl scaffold to improve its inhibitory efficacy toward SIRT5. Among the synthesized molecules, the (*S*)-3-(2-naphthylthio) succinyl derivative **1g** (Figure 5) displayed strong inhibition of SIRT5 deglutarylase activity, with an IC_50_ value of 30.3 nM and a *K*_*i*_ value of 30.1 nM.^150^ Consistent with this data, the zSIRT5–**1g** cocrystal structure showed that the naphthyl moiety completelyoccupied the C-pocket. Compound **1g** was also selective over other SIRT isoforms (SIRT1–3 and SIRT6) at concentrations up to 50 μM. To create a more drug-like structure, Kalbas et al. prepared compound **1h**, a tripeptide with the same 3-substituted succinyl scaffold discussed above. Although it is less potent than the parent compound, it still retains promising inhibitory activity, with IC_50_ = 350.4 nM and a *K*_*i*_ = 179.8 nM (deglutarylation), thereby representing a good starting point for further development.^150^

**Figure 6:**
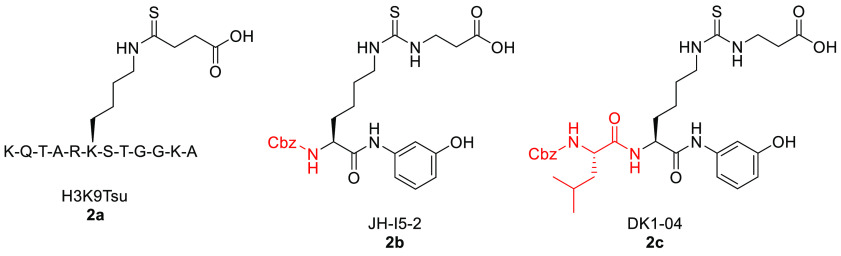
Structures of H3K9Tsu-derived peptide SIRT5 inhibitors **2a**–**c**.

With the aim of improving the cell permeability of SIRT5 inhibitors, Abril et al. modified a previously reported thiosuccinyllysine peptide (H3K9TSu, **2a**, Figure 6), which was found to inhibit SIRT5 desuccinylase activity with IC_50_ = 5 μM while being selective over SIRT1–3 (no inhibition at 100 μM). Indeed, they gradually shortened the peptide and replaced the thioamide moiety with a thiourea function to yield compound JH-I5-2 (**2b**), which consisted of a lysine derivative protected by a benzyloxycarbonyl (Cbz) group at the *N*-terminus and a *N*-(3-hydroxyphenyl) carboxamide moiety at the *C-*terminus.^135^ Despite the fact that the thiourea functionality is prone to metabolic *S*-oxidation *in vivo*, which is mostly mediated by cytochrome P450 and flavin-containing monooxygenases (FMO) causing the formation of sulfoxide intermediates that may also undergo hydrolysis to the corresponding urea,^151−154^ compound **2b** showed stronger SIRT5 inhibition, with an IC_50_ value of 2.1 μM for desuccinylation (Table 2). This potent inhibitory activity is probably due to the presence of the hydroxyl group on the *C*-terminal anilide moiety that provides an additional hydrogen bond, thereby granting tighter binding to the enzyme. DK1-04 (**2c**) was obtained by adding a Cbz-protected leucine residue to the *N*-terminus. This compound displayed the strongest inhibition of SIRT5 desuccinylase activity, with IC_50_ = 0.34 μM. Both **2b** and **2c** (Figure 6) showed selectivity for SIRT5, and no inhibition of SIRT1–3 or SIRT6 was detected at a concentration of 83.3 μM. These molecules are mechanism-based inhibitors that form a stalled covalent 1′-(*S*)-alkylimidate intermediate with the ADP-ribose in the active site, which blocks the catalytic mechanism. The group also synthesized two different pro-drug forms of these compounds to increase their cell permeability, which was compromised by the free carboxylic acid moiety. Hence, they developed **2b-*****am***, **2b-*****et***, **2c-*****am***, and **2c-*****et***, bearing an aceto-methoxy (*am*) or ethyl ester (*et*) group, which displayed cellular activity by increasing global lysine succinylation in MCF7 breast cancer cells at a concentration of 50 μM. **2c**-Based prodrugs significantly decreased MCF7 and MDA-MB-231 breast cancer cell viability (GI_50_(**2c-*****am***) = 51 μM and GI_50_(**2c-*****et***) = 20 μM). All compounds impaired the anchorage-independent growth of the same cell lines with GI_50_ values between 10 and 37 μM, although **2c**-based prodrugs were still more potent. In particular, the most effective prodrug was the ethyl ester derivative **2c-*****et***, which also blocked breast cancer growth in both genetically engineered and xenograft mouse models. In the case of genetically engineered mice, **2c-*****et*** was administered at a dose of 50 mg/kg five times per week for six weeks, while in the case of xenograft mouse models it was administered at the same dose for three weeks.^135^

**Figure 7:**
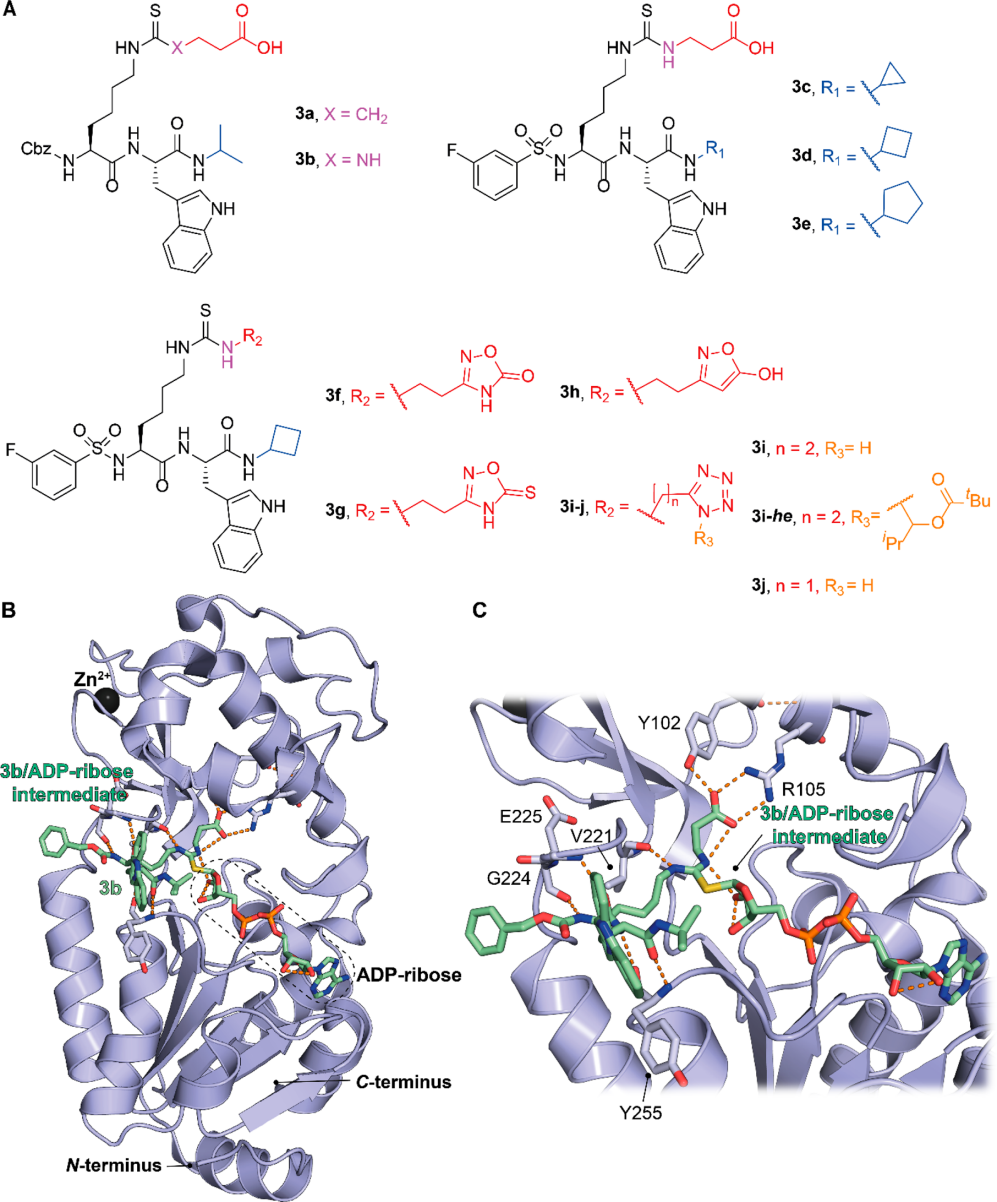
(A) Structures of ε-*N*-thioglutaryllysine derivatives **3a**–**j**. (B) Structure of hSIRT5 in complex with the ADP-ribose-1′-thioimidate intermediate of compound **3b** (green) (PDB ID 6EQS). (C) Focus on the binding site to show how the most important residues mediate the protein–compound interaction. Dashed orange lines indicate polar interactions.

Recently, Rajabi and colleagues developed a series of ε-*N*-thioglutaryllysine derivatives and performed an extensive SAR study to elucidate the molecular features necessary for SIRT5 inhibition.^155^ Compound **3a** is a submicromolar inhibitor of SIRT5 deglutarylase activity (IC_50_ = 0.83 μM) bearing a thioamide moiety, a Cbz-protected *N*-terminus, and a *C*-terminal *L*-Trp; **3a** has stronger inhibitory activity than the corresponding derivative with *D*-Trp (90% inhibition at 100 μM).^155^ Compound **3b** is a thiourea analogue of **3a** with IC_50_ = 0.37 μM for SIRT5-mediated deglutarylation (Table 2). Both **3a** and **3b** (Figure 2) are Cbz-protected at the *N*-terminus (Figure 7A). The research team managed to cocrystallize these two molecules with both human and zebrafish SIRT5 and confirmed the formation of a catalytic intermediate with the ADP-ribose and key interactions with residues Tyr102 and Arg105 (Figure 7B and C). Due to the lack of specific interactions of the benzyloxycarbonyl group, they investigated other structures, which led to the development of more derivatives bearing the same scaffold as **3a** and **3b** with different substitutions on the *N*- and *C*-termini. This led to compounds **3c**–**3e** (Figure 7A), which possessed a 3-fluorobenzensulfonamide at the *N*-terminus but differed at the *C-*terminus due to the substitution of the carboxamide N with a cyclopropyl, cyclobutyl, or cyclopentyl group, respectively. Among them, **3d** is the most potent SIRT5 inhibitor with an IC_50_ value 0.11 μM for deglutarylation (another study reported an IC_50_ value of 0.44 μM)^86^ (Table 2), while compounds **3c** and **3e** present IC_50_ values of 0.26 and 0.23 μM, respectively, against SIRT5 deglutarylase activity. As mentioned above, these compounds are mechanism-based inhibitors that promote the formation of a covalent stalled intermediate with NAD^+^ within the active site. Hence, using only IC_50_ values as indication of inhibitory potency may be erroneous, as they cannot be compared to those obtained with reversible inhibitors that are based on measurements at equilibrium. Nonetheless, the authors also obtained *K*_*i*_ values from continuous flow experiments for the most promising molecules, which enabled a kinetic analysis and a more accurate estimation of the inhibitor potency. Specifically, **3a**, **3b** and **3d** were shown to have a slow tight-binding mechanism of inhibition, and their *K*_*i*_ values are 22, 37, and 6 nM, respectively. In addition, compounds **3b**–**3e** showed great selectivity for SIRT5 over SIRT1–3 and 6, while **3a** was not tested against other isoforms.^155^ Among these molecules, **3b** and **3d** were subsequently tested in cellular assays as pro-drug esters. Indeed, to improve their cell permeability, the negatively charged carboxylic moiety was masked with an ethyl ester, yielding prodrugs **3b-*****et*** and **3d-*****et***. **3b-*****et*** and **3d-*****et*** were tested in AML cell lines whose proliferations were either SIRT5-dependent (OCI-AML2 and SKM-1) or SIRT5-independent (KG1a and Marimo). Both molecules inhibited cell proliferation and induced the apoptosis of SIRT5-dependent cells, while they did not show any effect on SIRT5-independent AML cell lines. Among the two molecules, **3b-*****et*** was the most potent one, with IC_50_ values of 5–8 μM, while **3d-*****et*** showed IC_50_ values of 10–20 μM. Accordingly, **3b-*****et*** induced more than 80% apoptosis at 5 or 10 μM in SKM-1 or OCI-AML2, respectively, while **3d*****-et*** induced more than 80% apoptosis only at 20 μM in SKM-1 (Table 2). Notably, the effects induced by **3b-*****et*** resembled SIRT5 knockdown. In addition, mice injected with **3b-*****et***-treated AML cells (at 12.5 or 25 μM) displayed higher survival rates compared to the controls.^147^

Interestingly, compound **3d** was recently tested in a SARS-CoV-2 infection cellular model.^86^ Initial experiments performed in HEK-293 SIRT5 knockdown cells transfected with SIRT5 and Nsp14 from SARS-CoV-2 indicated that this compound was able to disrupt the SIRT5–Nsp14 interaction starting at a concentration of 25 μM. More importantly, Calu-3 cells infected with SARS-CoV-2 and treated with **3d** displayed reduced viral titers and mRNA levels at 25 and 100 μM, respectively.^86^

Starting from compound **3d**, Rajabi et al. recently developed a series of derivatives to investigate whether the bioisosteric substitution of the carboxylic acid moiety might retain the SIRT5 inhibitory potency.^148^ Among the derivatives, 1,2,4-oxadiazol-5(4*H*)-one (**3f**), 1,2,4-oxadiazol-5(4*H*)-thione (**3g**), 2-hydroxyisoxazole (**3h**), and tetrazole (**3i**) displayed submicromolar IC_50_ values for SIRT5-mediated deglutarylation (IC_50_(**3f**) ≤ 0.05 μM, IC_50_(**3g**) = 0.9 μM, IC_50_(**3h**) = 0.29 μM, and IC_50_(**3i**) ≤ 0.05 μM). The kinetics of SIRT5 inhibition by compounds **3f**, **3h**, and **3i** was also evaluated. **3f** and **3i** exhibited *K*_i_ values in the low nanomolar range for (7 and 0.5 nM, respectively), while **3g** exhibited a *K*_i_ value of 122 nM. All compounds were also tested against SIRT1–3 and SIRT6 and displayed negligible inhibitory activities at 10 μM, with only **3f** displaying 37% SIRT1 inhibition and **3h** showing 40% SIRT3 inhibition at the same concentration. Notably, compound **3j** in which the alkyl spacer length was reduced to one methylene unit displayed a drop in potency (IC_50_ = 5.1 μM), thereby indicating the importance of both the length and the flexibility of the lysine side chain for the SIRT5 affinity of the isosteres (Figure 1), as previously shown for carboxylates.^76,155^ All newly developed compounds displayed poor cell permeabilities, which were comparable to that of the parent molecule **3d** and one order of magnitude lower than that of its ethyl ester **3d*****-et***. Hence, the authors prepared compound **3i*****-he***, a prodrug of **3i** bearing a masked tetrazole moiety, using an *O*-*tert*-butyloxycarbonyl-*N*,*O*-isobutyl hemiaminal functionality. Compound **3i*****-he*** was assessed for its *in vitro* activity toward SIRT1–3 and SIRT6 and presented 76% SIRT1 inhibition at 1 μM, showing that this masking group decreased the isoform selectivity compared to the unprotected parent molecule.

Cellular target engagement was then assessed in HEK293T cells for increasing compound concentrations (2.6 nM to 10 μM) of **3d**, **3d*****-et***, **3f**, **3g**, **3i**, and **3i*****-he*** via an isothermal dose–response fingerprinting cellular thermal shift assay (ITDRF-CETSA) performed at a constant temperature of 52 °C. Compounds **3f** and **3g** showed poor target engagement, with EC_50_ values higher than 10 μM, while compounds **3d** and **3i** exhibited EC_50_ values of 0.9 and 1.3 μM, respectively. Notably, the prodrugs **3d-*****et*** and **3i-*****he*** bearing masked acidic groups displayed more prominent target engagement, with EC_50_ values of 0.25 and 0.15 μM, respectively. Full melting experiments with **3i-*****he*** (1 μM) against SIRT1, SIRT3, and SIRT5 confirmed that target engagement and suggested selectivity over the other mitochondrial isoform SIRT3, with shifts in the protein melting temperature of 5.4 °C for SIRT5 and 0.5 °C for SIRT3. However, considerable engagement was observed for SIRT1, with a shift in the protein melting temperature of 4.6 °C, thereby suggesting the incomplete hydrolysis of the masking group inside HEK293T cells. Hence, more advanced masking approaches would be necessary to improve the selectivities of the tetrazole-containing derivatives.

When tested in SIRT5-dependent SKM-1 AML cells and immortalized HEK293T cells, compounds **3d**, **3f**, and **3i** did not exhibit any decrease in viability at concentrations up to 100 μM. Conversely, the **3d*****-et*** and **3i*****-he*** displayed IC_50_ values against SKM-1 cells of 21 and 9 μM, respectively. When tested in HEK293T cells, **3d*****-et*** displayed a IC_50_ value between 50 and 100 μM, while **3i*****-he*** showed a IC_50_ value higher than 100 μM with less than 35% growth inhibition at 100 μM, thereby indicating the higher cancer selectivity of **3i*****-he*** compared to **3d*****-et***. **3d*****-et*** and **3i*****-he*** were also assessed in two further SIRT5-dependent AML cell lines, OCI-AML2 and MOLM-13. **3i*****-he*** displayed a higher efficacy in OCI-AML2 (IC_50_(OCI-AML2, **3d*****-et***) > 50 μM and IC_50_(OCI-AML2, **3i*****-he***) = 20 μM), while similar cell growth inhibition was observed for MOLM-13 (IC_50_ (MOLM-13, **3d*****-et***) = 29 μM and IC_50_ (MOLM-13, **3i*****-he***) = 24 μM).^148^

**Figure 8:**
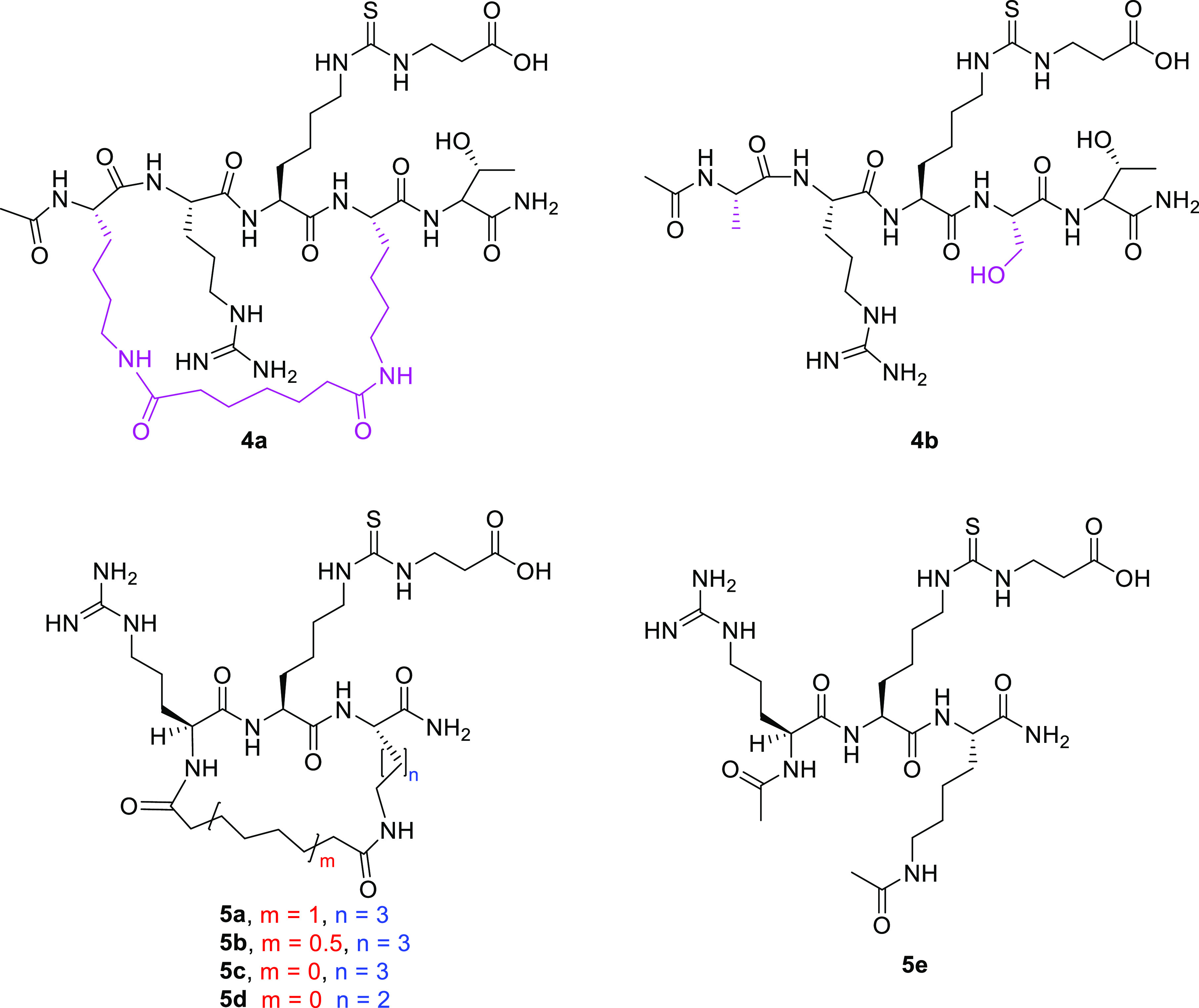
Structures of cyclic penta- and tripeptide SIRT5 inhibitors.

Given the potency and selectivity of the thiourea-type warhead, which can also circumvent the cytotoxicity issue that results from the thioamide-based derivatives, Liu and colleagues developed cyclic pentapeptides harboring a central ε-*N*-carboxyethylthiocarbamoyllysine residue. Compound **4a** (Figure 8), the side chain-to-side chain cyclic pentapeptide depicted in Figure 3, inhibits SIRT5 desuccinylase activity with IC_50_ = 7.5 μM and is selective over SIRT1–3 and SIRT6 (IC_50_ values >1 mM).^156^ Compared to its linear counterpart **4b** (Figure 8), compound **4a** was found to be more proteolytically stable when tested in proteolytic digestion using Pronase as the protease. In addition, compound **4b** was tested under the same SIRT5 inhibition assay conditions and was found to exhibit a SIRT5 inhibitory potency comparable to that of **4a** with an IC_50_ value of 7.6 μM (desuccinylase). However, it also exhibited a notable inhibitory activity against SIRT2 (IC_50_ = 96.4 μM) while still being selective over SIRT1, SIRT3, and SIRT6. These data suggest that this macrocyclic bridging unit is not favorable for enhancing the SIRT5 inhibitory potency compared to its linear counterpart and does not provide a tighter binding at the enzyme active site. Nonetheless, the presence of a macrocycle confers a better selectivity profile and greatly increases the metabolic stability. Hence, the macrocycle bridging unit immediately surrounding the warhead could serve as a lead for the development of new, more potent, and selective SIRT5 inhibitors.^156^ In line with this, in another study the same group synthesized a series of *N*-terminus-to-side chain cyclic tripeptides bearing the same SIRT5 inhibitory warhead as seen in the previous work, with the idea that the various bridging units would ensure a favorable interaction in the active site and yield tighter binding to SIRT5.^157^ Compounds **5a**–**d** (Figure 8) present spacers of various lengths between the *N*-terminal α-amino group and the side chain ε-amino group of the lysine residue and harbor an arginine residue at the *N*-terminus. Among them, compound **5c**, which presents a succinyl bridging unit, exhibited the greatest SIRT5 inhibitory activity with IC_50_ = 2.2 μM, being 2–6× more potent than compounds **5a**, **5b**, and **5d** (IC_50_(**5a**) = 13.2 μM, IC_50_(**5b**) = 6.5 μM, and IC_50_ (**5d**) = 4.0 μM; all values were measured using the succinyllysine SIRT5 substrate). Compound **5c** also displayed >60-fold selectivity over SIRT1–3 and SIRT6. Furthermore, compound **5e**, the linear counterpart of **5c**, exhibited a more than 42-fold decrease in SIRT5 inhibition (IC_50_(desuccinylation) = 93.1 μM), suggesting that in this case the peptide chain macrocyclization could enhance the target binding affinity. A proteolysis assay performed using the Pronase as proteolytic enzyme again indicated the higher proteolytic stability of the cyclic peptide **5c** compared to its linear counterpart **5e**. In conclusion, the tripeptide **5c** displayed a SIRT5 inhibitory potency more than threefold greater than the previously reported pentapeptide **4a**, suggesting that this smaller peptide could be a useful starting point for further SAR investigations to obtain new, more potent, and selective SIRT5 inhibitors.^157^

**Figure 9:**
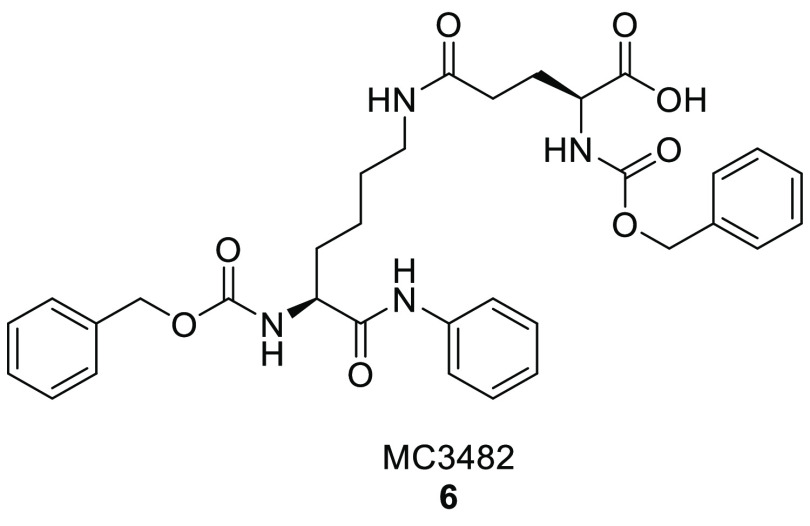
Structure of MC3482.

Polletta et al. recently developed MC3482 (**6**, Figure 9), an ε-*N*-glutaryllysine-based compound wherein the α-amine of the lysine residue is Cbz-protected while the C-terminal carboxy group forms an anilide function.^84^ Compound **6** was reported to be a promising inhibitor of SIRT5-mediated desuccinylation, exhibiting dose-dependent activity and reaching 42% SIRT5 inhibition at 50 μM when tested in MDA-MB-231 cells without having effect on SIRT1 and exhibiting only 8% SIRT3 inhibition at the same concentration. Moreover, both human breast cancer cells (MDA-MB-231) and mouse myoblasts (C2C12) treated with compound **6** (50 μM) displayed an increase in succinylated proteins as a result of the inhibition of SIRT5 desuccinylase activity.^84^ In addition, treating MDA-MB-231 and C2C12 cells with compound **6** (50 μM) led to an increase in cellular glutamate and ammonia levels via an increase GLS succinylation. These results are in line with SIRT5’s role in the regulation of ammonia production through the modulation of glutamine metabolism. In the same report, compound **6** was also shown to promote ammonia-induced autophagy and mitophagy (Table 2). In a recent study, Molinari and co-workers demonstrated that this compound was also able to stimulate the expression of brown adipose tissue markers, thus facilitating preadipocyte differentiation into brown-like adipocytes when dispensed at early stages of differentiation.^158^ Furthermore, treatment with compound **6** at 50 μM led to more efficient mitochondrial activity and biogenesis along with a higher lipolytic rate associated with an increase of triglyceride lipase expression, indicating that SIRT5 inhibition is a favorable strategy to treat obesity and metabolic diseases.^158^

#### 4.1.2 Small-Molecule Inhibitors

**Figure 10:**
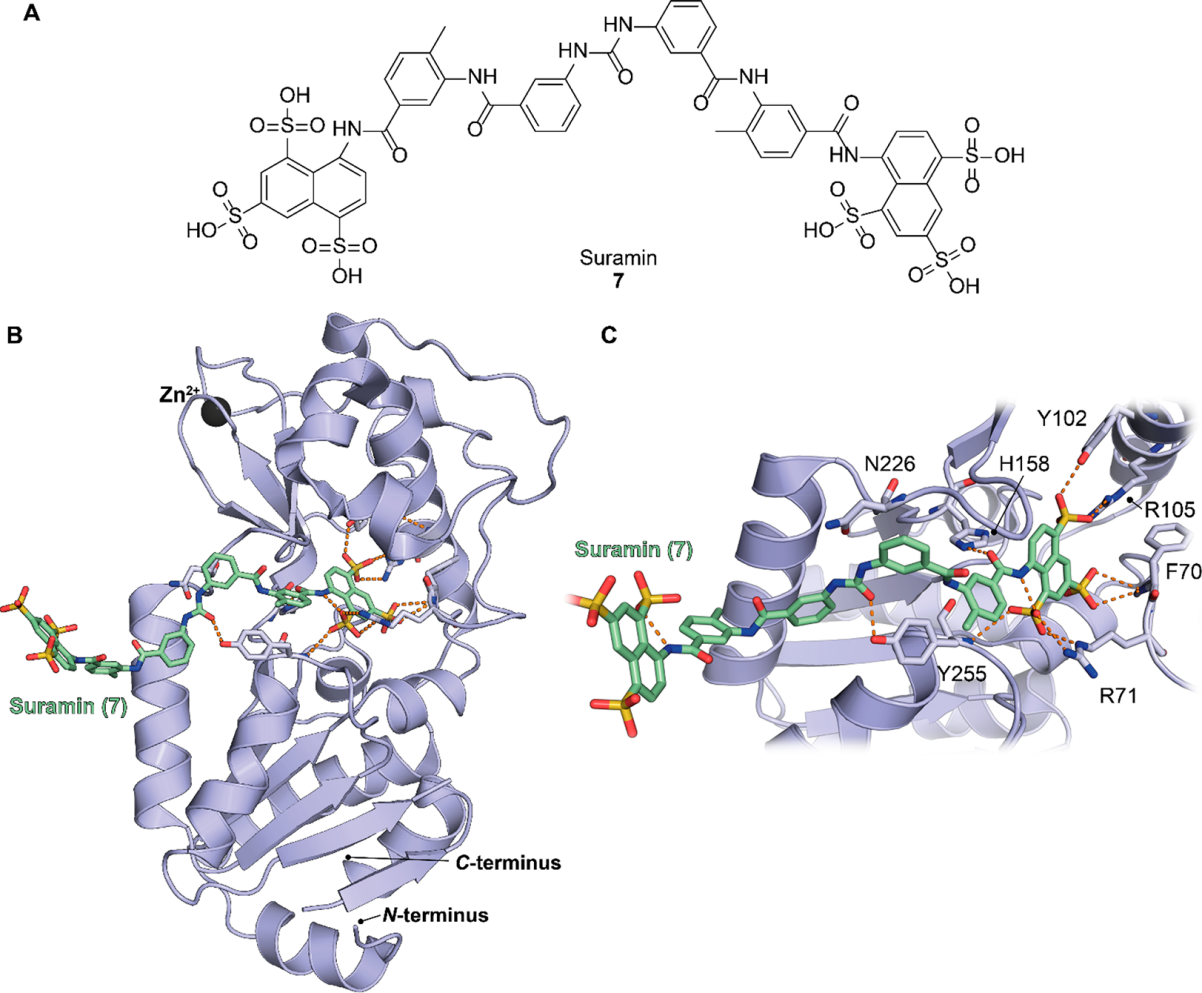
(A) Structure of suramin (**7**). (B) Structure of hSIRT5 in complex with compound **7** (green) (PDB ID 2NYR). (C) Focus on the binding site of compound **7** showing how the key residues mediate the protein-compound interaction. Dashed orange lines indicate polar interactions.

Suramin (**7**, Figure 10A), a well-known antiparasitic agent, was identified as one of the first sirtuin inhibitors and found to also inhibit SIRT5.^159^ To comprehend how this molecule binds to the enzyme and the structural and molecular mechanisms of inhibition, Schuetz et al. determined the crystal structure of SIRT5 in complex with **7**. Interestingly, SIRT5 dimerizes in solution upon suramin binding and is stabilized by the suramin itself. The main interactions with the enzyme originate from the sulfonate groups of **7**, which form hydrogen bonds with the side chains of Arg71, Tyr102, Arg105, and Arg141 and with the backbone amide of Phe70 (Figure 10B and C). Interestingly, Phe70 and Arg71 seem to have a role in the release of nicotinamide, thereby suggesting that **7** mimics this reaction product when interacting with SIRT5. Tyr102 and Arg105 are also involved in interactions with the acyl-lysine substrate, thus suggesting that suramin occupies the peptide’s substrate-binding site. Furthermore, the carbonyl oxygen of the amide portion that connects the naphthalene to the benzene moiety of **7** forms a hydrogen bond with His158 (Figure 10B and C), thereby mimicking the interaction between the 3′-hydroxyl group of NAD^+^. This was confirmed by the superimposition of the SIRT5–ADP-ribose and SIRT5–suramin complex structures that showed **7** occupied the C-pocket, thus indicating that suramin mimics the binding of the cosubstrate. In addition, the central urea portion connecting the two symmetric portions of compound **7** forms a hydrogen bond with the hydroxyl group of Tyr255 (Figure 10B and C), which is usually involved in peptide substrate binding. Collectively, these results suggest that suramin inhibits SIRT5 activity through various interactions in the active site, as it resembles the interactions of substrate, product, and cosubstrate.^159^

The evidence that **7** interacts with the NAD^+^-binding site makes it nonselective over other isoforms possessing a similar cosubstrate binding pocket. In fact, it not only inhibits SIRT5 NAD^+^-dependent deacetylase activity with IC_50_ values of 14.2 and 22 μM,^159,160^ depending on the study, but also targets SIRT1 (IC_50_ = 0.297–2.6 μM, depending on the study)^159,161^ and SIRT2 (IC_50_ = 1.15 μM). A recent study also reported the **7**-mediated inhibition of SIRT5 desuccinylation activity, with IC_50_ = 46.6 μM.^162^ Overall, to overcome this lack of selectivity, it would be necessary to preferentially target the peptide substrate-binding site to avoid binding to other NAD^+^-dependent enzymes. Compound **7** was also tested in A549 lung cancer cells, where it seemed to increase the activity of PKM2, an enzyme inhibited by SIRT5-mediated desuccinylation, and lead to the suppression of cancer cell proliferation.^95^

With the aim of improving the knowledge of the isoform selectivity of potential new SIRT5 inhibitors and discovering the key interactions that lead to greater inhibition, Maurer and co-workers screened their internal library and found thiobarbiturates were potential SIRT5 inhibitors. This series of compounds (**8a**–**g**, Table 3) displayed inhibitions for SIRT5-mediated desuccinylation in the mid to low micromolar range, although they showed similar inhibitions for SIRT1 and 2 and displayed low potencies against SIRT3.^162^ Compound **8a**, bearing a 2-(2,3-dichlorophenyl)furanyl substitution, is a good SIRT5 inhibitor (IC_50_(SIRT5) = 3.6 μM); however, its selectivity profile is not ideal, since it inhibits SIRT1 with the same potency (IC_50_ (SIRT1) = 3.4 μM)) (Table 3). Derivative **8b**, bearing an allyl substitution at the thiobarbituric nitrogen, displays improvements in terms of potency and selectivity, with an IC_50_ value of 2.3 μM for SIRT5 (IC_50_(SIRT1) = 5.3 μM, IC_50_ (SIRT2) = 9.7 μM, and 41% inhibition of SIRT3 at 50 μM, Table 2). Notably, compound **8b** is the most potent and selective among the reported molecules. Conversely, the same allyl substitution is detrimental for compound activity in the series of compounds **8c**–**h** bearing a benzyloxyphenyl substitution, as indicated by the high IC_50_ value of compound **8f** (Table 3). In contrast, the alkyl substitution improves the selectivity over SIRT1 but not SIRT2 and SIRT 3, as indicated by the IC_50_ values of compounds **8d** and **8e** compared to those of **8c**, **8g**, and **8h**, which are unsubstituted on the thiobarbituric nitrogen (Table 3).

**Table 3:**
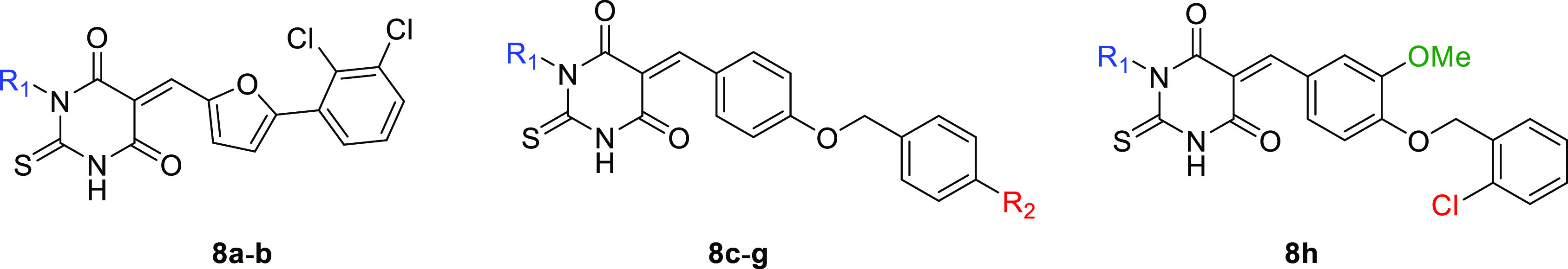
Structures and Inhibition Data of Thiobarbiturates **8a**–**g**^a^

			IC_50_ (μM)			
compd	R_1_	R_2_	SIRT1	SIRT2	SIRT3	SIRT5
**8a**	–H		3.4	10.5	30% inhib. @ 50 μM	3.6
**8b**	—CH_2_CH=CH_2_		5.3	9.7	41% inhib. @ 50 μM	2.3
**8c**	–H	–H	10.5	9.8	29.3	12.6
**8d**	–CH_3_	–H	56.5	10.0	22% inhib. @ 50 μM	17.8
**8e**	-–H_2_CH_3_	–H	53.2	14.4	25% inhib. @ 50 μM	12.9
**8f**	—CH_2_CH=CH_2_	–H	26.8	no inhib. @ 50 μM	13% inhib. @ 50 μM	67.3
**8g**	–H	–Br	9.9	3.4	30.3	6.2
**8h**	–H		6.7	7.5	46.4	12.4

a SIRT5’s IC_50_ values were measured against its desuccinylase activity.

Overall, these results indicate that the 2-(2,3-dichlorophenyl)furanyl substitution is more favorable than the benzyloxyphenyl one. The research team also performed docking studies to characterize the interactions of the compounds within the active site of SIRT5. They found that the thiobarbiturate ring fits into the substrate-binding site and forms hydrogen bonds with Tyr102, Arg105, and Gln140, thus mimicking the substrate succinyl group. In particular, such interaction is stabilized by strong electrostatic contacts between the basic guanidinium group of Arg105 and the acidic thiobarbiturate.^162^ Two related thiobarbiturates were recently identified as non-nucleoside inhibitors of the H3K79 histone methyltransferase DOT1L using ligand-based and structure-based combined approaches, thus suggesting the promiscuous nature of these compounds.^163^

Starting from a virtual screening aimed at finding novel SIRT5-selective inhibitors, Liu and colleagues initially identified compounds **9a** and **9b** (Table 4), which inhibited SIRT5-mediated desuccinylation with IC_50_ values of 18.30 and 9.26 μM, respectively.^164^ According to docking studies, the carboxylate group of the two compounds forms hydrogen bonds and electrostatic interactions with Tyr102 and Arg105. To improve the inhibitory potency and identify the structural features key to the activity, various **9b** analogues were prepared, all of which had the same central (*E*)-2-cyano-*N*-phenyl-3-(5-phenylfuran-2-yl)acrylamide core with different substitutions at the amide (moiety **A**) and at C5 furan ring position (moiety **B**). According to the IC_50_ measurements, for moiety **B**, the *para*-benzoic acid substitution is preferred to the *meta*-benzoic acid substitution, and a further alkyl substitution on the phenyl ring of the *para*-benzoic acid is detrimental (see compounds **9c**–**e**, Table 4). Hence, the presence of a carboxylic acid at the *para*-position most likely provides the right orientation for the compound to interact with residues Tyr102 and Arg105 in the active site of SIRT5. Regarding moiety **A**, the presence of electron-withdrawing groups (EWGs) at the *meta*- and *para*-phenyl ring positions seemed to increase the inhibitory potency, as suggested by the lower IC_50_ values of **9f**–**i** compared to those of **9c** and **9j** (Table 4). The most potent compound, **9g** (IC_50_ = 5.59 μM), was also assessed against SIRT2 and SIRT6, where it displayed no inhibition up to 600 μM (Table 2). Furthermore, its inhibitory activity was not affected by the NAD^+^ concentration, suggesting that **9g** is not competitive toward NAD^+^ but acts via competitive inhibition with the succinyl-lysine substrate. The docking analysis indicated that it likely forms hydrogen bonds with the side chains of Tyr102 and Arg105 and backbone amides of Leu227 and Try255, with the fluorine atom forming a halogen bond with Asn226.^164^

**Table 4:**
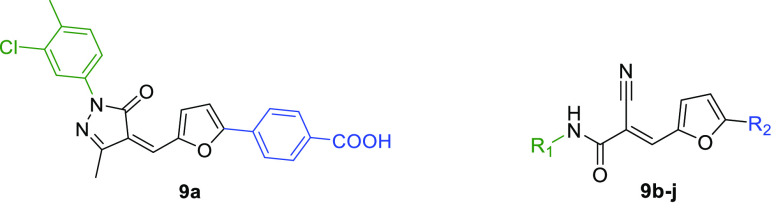

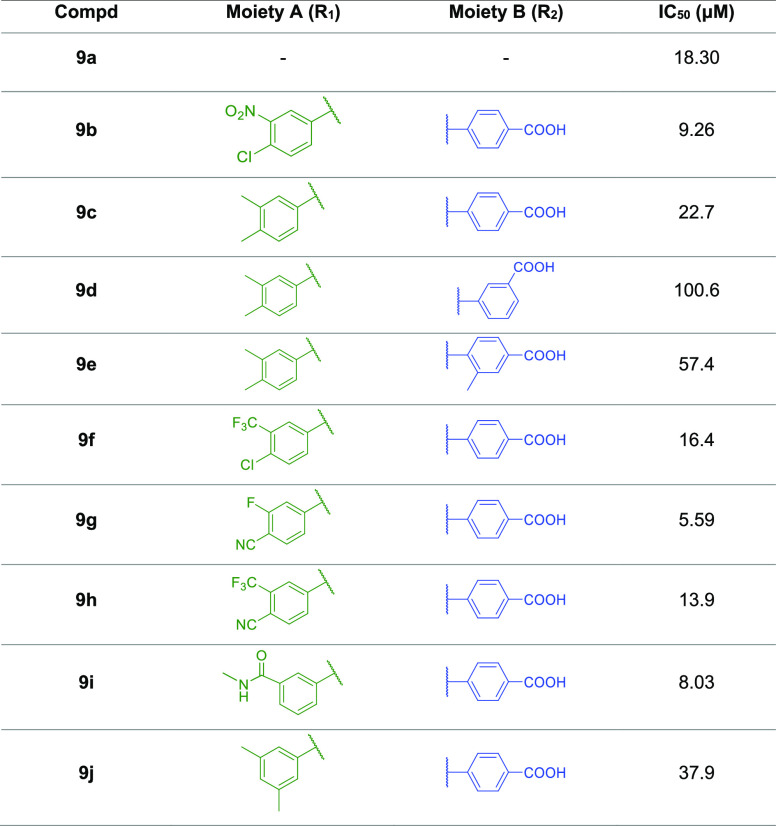
Structures and IC_50_ Values (μM) for the Desuccinylase Activity of SIRT5 toward (*E*)-2-Cyano-*N*-phenyl-3-(5-phenylfuran-2-yl)acrylamide Derivatives

**Figure 11:**
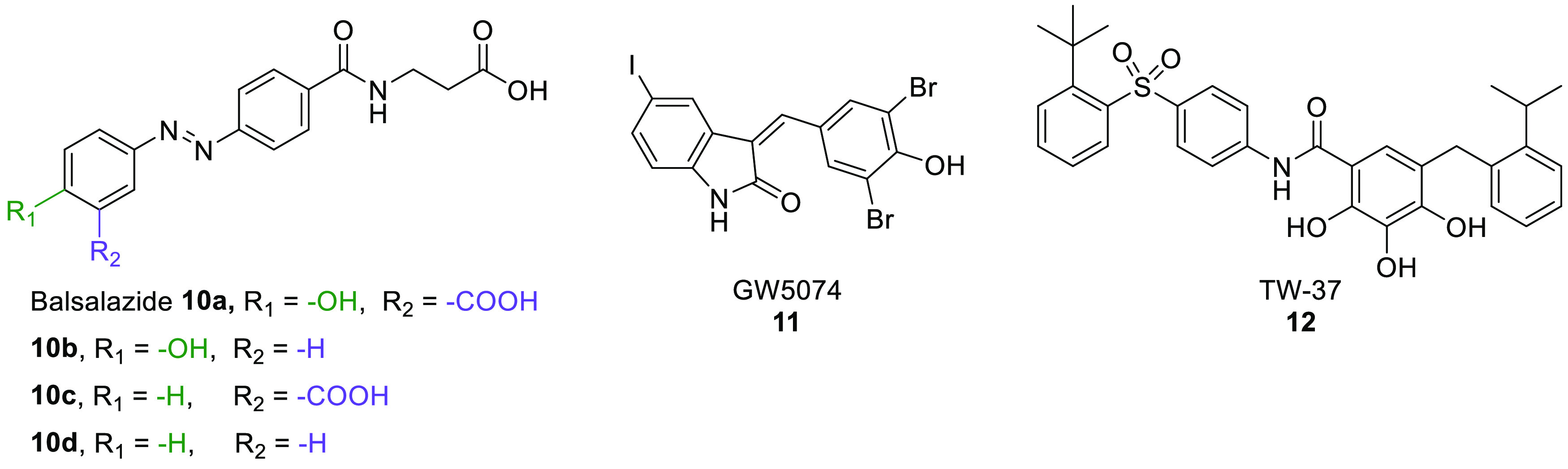
Structures of compounds **10a**–**d**, **11**, and **12**.

In the search for selective inhibitors, Guetschow et al. carried out a high-throughput screening of a library of 1280 compounds using microchip electrophoresis and found 8 molecules able to inhibit SIRT5’s desuccinylating activity.^165^ Among them, balsalazide (**10a**, Figure 11) was reported to have an IC_50_ value of 3.9 μM. Compound **10a** is an approved nonsteroidal anti-inflammatory drug currently employed for the treatment of inflammatory bowel disease. This molecule presents a salicylic moiety that is connected by a central azo group, with a benzamide substituted with a β-alanine side chain. To gain more insights into the binding mode of **10a** and explore the possibility of further optimization, Glas and colleagues set out to perform a SAR study using **10a** as the lead compound.^166^ They initially performed docking calculations for **10a** bound to a previously reported SIRT5–succinyl-lysine-based peptide cocrystal structure (PDB ID 3RIY)^69^ in the presence of NAD^+^. They found that **10a** not only forms hydrogen bonds with Tyr102 and Arg105 residues through its carboxylate group but also forms hydrogen bonds with a hydroxyl group of the cosubstrate NAD^+^ and with the backbone residues Val221 and Glu225 via its amide moiety. These results suggest that the side chain of **10a**, derived from β-alanine, is likely the moiety that contributes to the affinity and thus the inhibitory effect of **10a**, while the role of the salicylic group remains to be assessed. With the aim of investigating which functional groups of **10a** were essential for its inhibitory activity, the research group synthesized a series of 13 analogues.^166^ The initial evaluation of **10a** toward SIRT5’s desuccinylation activity yielded an IC_50_ value of 5.3 μM and 83% inhibition at 50 μM (Table 2). Removing functional groups from the salicylic portion of balsalazide yielded the phenol derivative **10b**, the benzoic acid derivative **10c**, and the phenyl analogue **10d** (Figure 11), which displayed 73%, 63%, and 62% inhibition of SIRT5’s desuccinylation activity at 50 μM, respectively. Conversely, removing the carboxamide moiety led to compounds with reduced inhibitory activities (30% or lower at 50 μM). These results confirm the hypothesis that the carboxylate group of the β-alanine side chain is crucial for the interactions in the active site, while modifications in the salicylic acid moiety are partially tolerated. In addition, these compounds were tested against the other SIRT isoforms at 50 μM and showed very low inhibitory activities, thereby showing they were SIRT5-selective. Furthermore, the authors indicated that **10a** and **10b** do not compete with the cosubstrate NAD^+^ or the synthetic substrate ZKsA. Unfortunately, **10a** hardly solubilizes in water and it is likely to be hydrolyzed through enzymatic degradation, hence it can not be used as a possible drug to target SIRT5.^166^

Suenkel et al. tested a series of previously reported SIRT inhibitors for their effects on SIRT5 deacetylation and desuccinylation.^160^ Among them, GW5074 (**11**, Figure 11), an indole derivative previously reported as Raf-1 kinase (IC_50_(Raf-1) = 9 nM)^167^ and a SIRT2 inhibitor (IC_50_(SIRT2) = 12.5 μM),^168^ inhibited SIRT5 desuccinylation activity with an IC_50_ value of 19.5 μM using a succinylated peptide derived from peroxiredoxin 1 (succPrx1). Interestingly, when tested at the same concentration range for its inhibitory effects on SIRT5 deacetylation activity using acPrx1, **11** showed an IC_50_ value of about 200–400 μM. These data indicate that acPrx1 is a weak SIRT5 substrate and that only **11** inhibits SIRT5 desuccinylation significantly. When a different acetylated peptide (acCPS1) was used, **11** inhibited SIRT5 deacetylation activity with an IC_50_ of 97.8 μM, which is still fourfold higher than the IC_50_ for the inhibition of SIRT5’s desuccinylation activity. Collectively, these results suggest that both the substrate sequence and the acyl modification influence the compound inhibitory potency.^160^ Compound **11** was also tested in the CRC cell line HCT116, where it reduced SIRT5 levels, consequently decreased the activity of its substrate LDHB, and finally decreased autophagy and cell proliferation.^142^ Notably, **11** showed similar results in terms of its influence on SIRT5 expression, autophagy, and tumor growth in mouse xenograft models. Overall, although promising, this study did not demonstrate that the effects of **11** are a consequence of SIRT5 inhibition but rather indicated that it was able to modulate its expression.

Yang et al. reported 16 fluorogenic peptide SIRT substrates that were tested against SIRT isoforms to determine their sensitivity and efficiency through fluorescence-based assays used to identify SIRT inhibitors.^79^ Since three succinyl-modified substrates showed high sensitivities and selectivities for SIRT5, three of them were cocrystallized with the enzyme. Crystallographic analyses revealed that these peptides placed the succinyl-lysine moiety in the substrate-binding site of SIRT5, forming hydrogen bonds with residues Tyr102, Arg105, Val221, Gly224, and Glu225 and π–π stacking interactions with the residues Leu227, Met259, Asn226, and Tyr255. These promising substrates led the authors to perform an *in-house* library screening, which identified TW-37 (**12**, Figure 11), an inhibitor of Bcl-2 family members, as a SIRT5 inhibitor. Docking studies indicated that **12** binds into SIRT5 substrate-binding pocket as well as the C-pocket. The IC_50_ values against SIRT5 were determined using three of the peptide substrates previously developed and were 21.9, 6.6, and 6.1 μM. In addition, compound **12** displayed no inhibition toward SIRT1–3.^79^ Hence, this molecule represents a new starting point for the development of dual SIRT5 and Bcl-2 inhibitors that may be relevant in cancer types where both proteins play a critical role.

Another study identified SIRT5 inhibitors by joining a heteroaromatic ring to a 3-thioureidopropanoic acid warhead through an aminoethyl linker to mimic the interactions of ε-*N*-glutaryllysine within the SIRT5 active site.^169^ Among the synthesized molecules, compounds **13a** and **13b** bearing a pyridine scaffold and a 2-benzylamino substitution, respectively, were about threefold less potent than compound **13c** (IC_50_(desuccinylation) = 9.6 μM), where pyridine was replaced by pyrimidine (Table 5). Starting from **13c**, various modifications were performed to gain SAR information. Increasing the length of the linker, as in the case of **13d** bearing a 2-phenetylamino substitution, led to a decrease in the potency. Conversely, replacing the nitro group at the C5 (R_3_) position of the pyrimidinyl ring with an ethoxycarbonyl group (**13e**) enhanced the inhibitory activity (IC_50_ = 3.0 μM), while the absence of a substitution (**13f**) or replacement with fluorine (**13g**) led to a drop in the inhibitory potency (Table 4), probably due to the lack of interactions with the active site. A similar trend was observed when the 2-benzylamino on the pyrimidinyl ring was replaced with a (2-(1*H*-indol-3-yl)ethyl)amino portion. Indeed, the C5-nitro derivative **13h** was about 3-4× less potent than **13i** and **13j**, which harbored ethoxycarbonyl and carboxylate substitutions at C5, respectively (Table 4). Interestingly, moving the carboxylate group from C5 to C4 (**13k**) led to a fourfold drop in inhibitory activity. These findings indicate that the presence of a carboxylate or ethoxycarbonyl moiety as R_3_ is favorable for compound activity. Furthermore, the most potent compounds (**13e**, **13i**, and **13j**) showed no inhibitory activities against SIRT1–3 or SIRT6 up to concentrations of 600 μM. The docking analysis of **13j** binding to hSIRT5 indicated that the carboxylate group forms hydrogen bonds and electrostatic interactions with Tyr102 and Arg105 as well as hydrogen bonds with Val221, Glu225, and Tyr255, thus suggesting that **13j** acts by mimicking the acyl-lysine substrate via a competitive mechanism of inhibition. Moreover, **13j** may react with the cosubstrate NAD^+^ to form a more stable ADP-ribose-1′-thioimidate intermediate.^169^

**Table 5:**
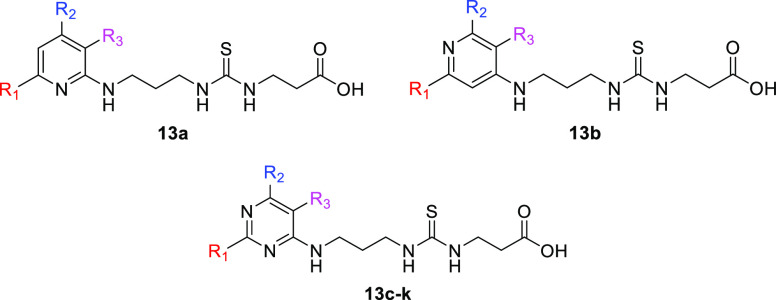

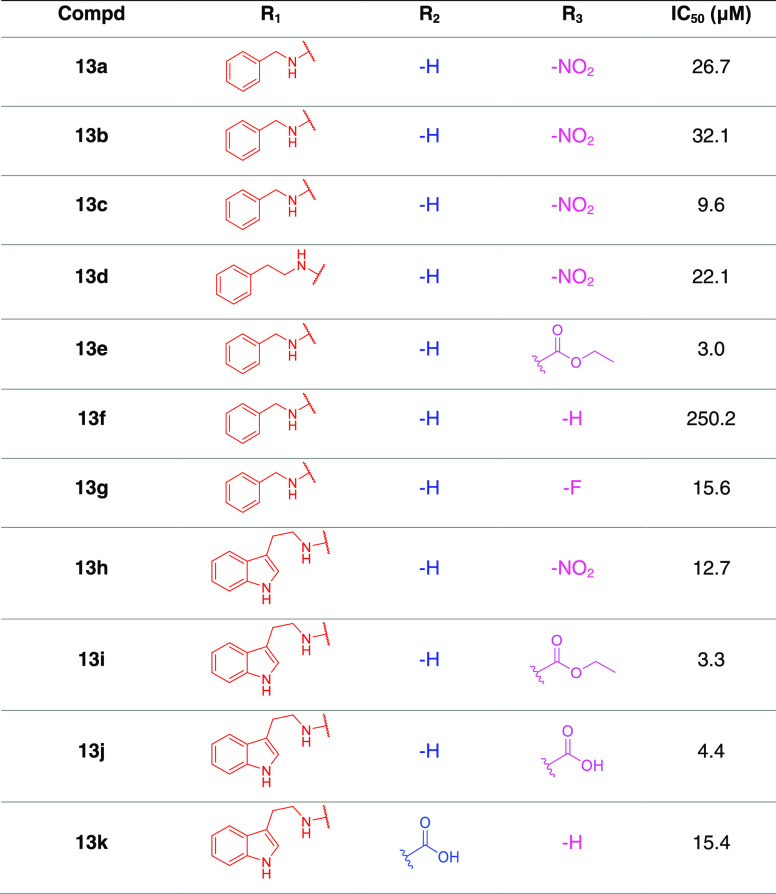
Structures and IC_50_ Values (μM) for the Desuccinylase Activity of SIRT5 toward 3-Thioureidopropanoic Acid Derivatives

### 4.2 SIRT5 Activator

**Figure 12:**
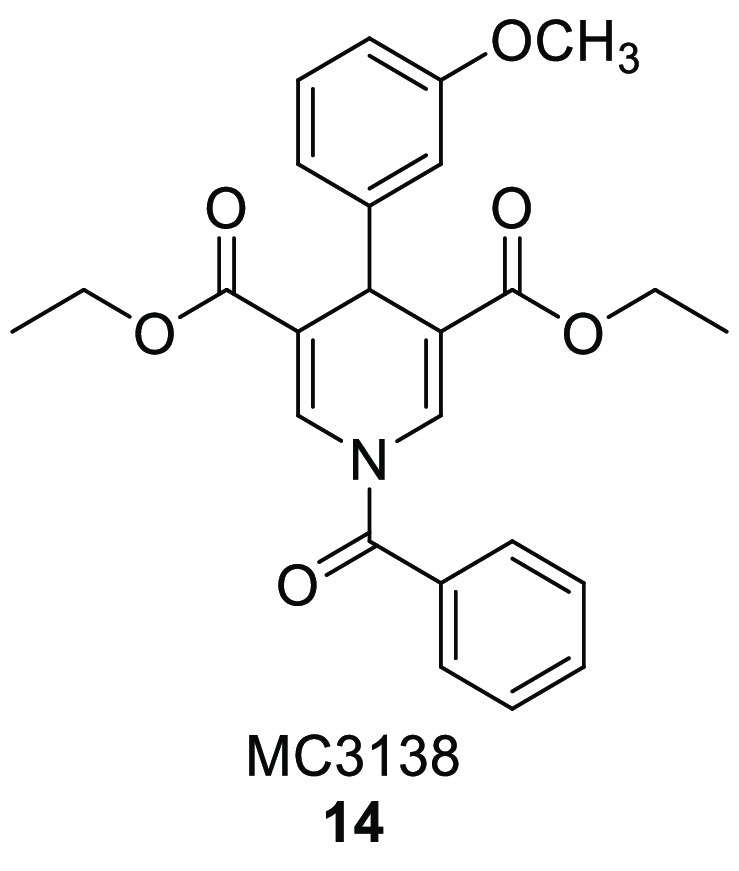
Structure of the SIRT5 activator MC3138 (**14**).

Although enhancing SIRT5 activity may benefit its role in human homeostasis, research in this field has been slower compared to SIRT5 inhibition, and only one small molecule has been described so far. Hu and colleagues recently reported the first small-molecule SIRT5 activator, termed MC3138 (**14**, Figure 12).^130^ This 1,4-dihydropyridine compound is structurally related to previously reported SIRT1 activators^170−172^ but displays selective SIRT5 activation, since it does not show any activity toward SIRT1 and SIRT3. Compound **14** increased SIRT5 deacetylase activity ∼1.5-fold at 10 μM, ∼3-fold at 50 μM, and ∼4-fold at 200 μM. Treating different PDAC cell lines with **14** led to a deacetylation profile like that caused by SIRT5 overexpression, resulting in the inhibition of GOT1 enzymatic activity. Compound **14** also decreased PDAC cell viability, with IC_50_ values ranging between 25.4 and 236.9 μM, and reduced metabolite levels involved in the glutamine, glutathione, and pyrimidine metabolic pathways. Selective SIRT5 activation at the cellular level was confirmed by experiments in the mouse PDAC cell lines KPC and KPCS. While KPC cells were sensitive to **14**, KPCS cells, which do not express SIRT5, were indeed resistant to compound **14** treatment, thereby indicating a causal correlation between SIRT5 activation and the anticancer properties of **14**. Furthermore, association of **14** and gemcitabine, an approved chemotherapeutic drug used for PDAC, resulted in synergistic effects at different concentrations in different human PDAC cell lines; this association also reduced tumor size and tumor weight *in vivo* and was well-tolerated in mice.

## 5 Conclusions

A growing body of research has shown that SIRT5 primarily performs protein deglutarylation, desuccinylation, and demalonylation at the cellular level and, given its main subcellular localization, preferentially targets mitochondrial proteins (see Figure 3 for an overview of proteins modulated by SIRT5). This is in line with the critical roles that SIRT5 has been shown to play in maintaining cellular homeostasis. These include regulating glycolysis, the TCA cycle, oxidative phosphorylation, FAO, ketone body formation, amino acid catabolism, and ROS management. SIRT5 enzymatic activities are particularly important for brain and heart health, particularly in the context of aging and in response to environmental and oxidative stress.

In the context of cancer, SIRT5 plays a dichotomous role, since it either suppresses or promotes cancer initiation or progression depending on various factors such as tissue or cell type and transformation stage. This is a consequence, at least in part, of the SIRT5-mediated regulation of ATP production and oxidative stress. Indeed, ROS detoxification may be beneficial in healthy cells to avoid DNA damage and prevent tumorigenesis. On the other hand, the same mechanism protects tumor cells from apoptosis, supports cell proliferation, and may reduce the susceptibility to genotoxic chemotherapeutics.

Given the manifold functions of SIRT5, potent and selective chemical tools that may act as either inhibitors or activators are urgently needed. Indeed, the selective modulation of SIRT5 may help researchers to further understand its roles in both physiological and pathological settings. In addition, potent and selective modulators may have the potential to be brought to the clinic to treat specific pathologies in which SIRT5 has a central position. Nonetheless, the road to the discovery of such modulators is at its infancy, in line with the fact that SIRT5’s activity and biological roles were validated quite recently compared to those of other sirtuins such as SIRT1.

Among SIRT5 inhibitors, a great deal of work has been carried out starting from peptide substrate analogues, which has led to promising peptide-based inhibitors. These consist of thiourea-based molecules such as **2c**,^135^**3b**, **3d**,^155^ and **3i**,^148^ which display submicromolar SIRT5 inhibition (Table 2). These molecules were all administered as prodrugs to mask the negative charge of the carboxylic group and increase cellular permeability. Compounds **2c** and **3b**, administered as ethyl esters, displayed very promising activities in breast cancer and AML cell lines, respectively. Moreover, **2c*****-et*** was also effective in both genetically engineered and xenograft mouse models of AML,^135^ while **3b-*****et*** was effective when administered to *ex vivo* AML cells subsequently injected in mice.^147^ Notably, compounds **3b*****-et***, **3d*****-et***, and **3i*****-he*** showed cellular effects only in SIRT5-dependent AML cell lines, thereby indicating a connection between SIRT5 inhibition and the observed anticancer effects. Nonetheless, further masking groups need to be explored to further increase cell permeability and avoid off-target effects such as in case of **3i*****-he***, which displays SIRT1 inhibition *in vitro* along with cellular target engagement. As for **2c*****-et***, although it determined cellular hypersuccinylation, further target engagement or genetic experiments would be necessary to confirm a causal correlation between SIRT5 inhibition and its phenotypic effects.

Regarding small-molecule SIRT5 inhibitors, there is still a great amount of research to be done. Indeed, only a few compounds have shown IC_50_ values in the low micromolar range. The 3-thioureidopropanoic acid derivative **13e** is the most potent and selective, with an IC_50_ value of 3.0 μM and no activity toward SIRT1–3 or SIRT6 at 600 μM.^169^ This molecule has been obtained in the context of a medicinal chemistry campaign aimed at finding glutaryl-lysine mimicking molecules. Unfortunately, no biological data have been provided, hence it is unclear whether this class of molecules may have any cellular effect and in which context they may be useful. Other inhibitors that displayed activities in the low micromolar range and isoform selectivity are **9g**(164) and balsalazide (**10a**)^166^ (Table 2). Nonetheless, **9g** has only been tested toward SIRT2 and SIRT6, while **10a** is an approved anti-inflammatory drug that acts as a prodrug and therefore possesses alternative modes of action beyond SIRT5 inhibition.^173^ Initial attempts to optimize **10a** in terms of its potency and pharmacokinetic properties failed, hence further development is still needed. Among small-molecule inhibitors, only the unselective compound **11** was assayed in cellular models and displayed some effects related to SIRT5 inhibition; however, these effects seemed mostly associated to modulation of SIRT5 levels, and a direct inhibitory effect was not proven.^142^

Among SIRT5 inhibitors, compounds **3b-*et***, **3d**, **6**, **7**, **9g**, **10a**, **11**, and **12** are commercially available, although it should be noted that compounds **3b-*****et***, **3d**, and **6**, are the only inhibitors that can be regarded as SIRT5-selective.

Nonetheless, more work would be necessary to deliver nanomolar inhibitors of SIRT5. To this end, cocrystal structures of SIRT5 in complex with currently known inhibitors provide valuable information for further drug development. In particular, the structures of hSIRT5 in complex with compound **3b** and **7** indicated the residues that should be targeted to develop an effective inhibitor. These include the key residues Tyr102 and Arg105, which are involved in substrate recognition, but also surrounding residues present in the catalytic cleft such as His150 and Tyr255, which interact with both **3b** and **7**.

The road to the release of potent SIRT5 activators is also still at its early stages, since only one molecule (**14**) has been described so far.^130^ Notably, this compound displays promising anticancer activity in PDAC cell lines as a consequence of SIRT5 activation and represents an optimal lead molecule for the development of a 1,4-dihydropyridine-based series of SIRT5 activators.

To date, the best lead structures for SIRT5 modulation are represented by the peptide-based inhibitors **2c-*****et***, **3b-*****et***, and ***3i-he*** and the 1,4-dihydropyridine activator **14**. Further efforts will be necessary to improve the potency, selectivity, and drug-likeness of currently available modulators. To this end, available cocrystal structures, along with high-throughput screening approaches, will likely aid the quick and reliable development of new chemotypes. For instance, the SIRT5–**3b**/ADP-ribose-1′-thioimidate intermediate structure could be used to develop new peptidomimetics in which the peptide groups are removed via isosteric substitution to decrease the peptide character of the molecule and nonessential side chains are modified to improve the pharmacokinetic properties. Moreover, to further increase the compound selectivity and potency, scaffold hopping^174^ strategies could be applied to yield new compounds bearing different cores while retaining the pivotal groups for SIRT5 binding.

In summary, although the field of SIRT5 modulation is still at its infancy, the availability of many SIRT5 crystal structures suggests that structure-based drug design approaches are possible. This strategy, in combination with modern computational and biophysical methods, bears great promise for the development of new molecules that could be used as valuable chemical probes for studying the biology or potential therapeutics of SIRT5.

More generally, the potential applicability of SIRT5 modulators in specific pathologies requires concerted efforts to gain a better idea of the roles of SIRT5 in different contexts and to clarify the relevance of its catalytic activity in both physiological and pathological states. This is particularly relevant in cancer, where SIRT5 may act as either tumor promoter or tumor suppressor even in the same cancer type.

## Abbreviations Used

ACAT1: acetyl-CoA acetyltransferase 1
ACOX1: acyl-CoA oxidase 1
AMPK: AMP-activated protein kinase
BAT: brown adipose tissue
Bcl-XL: B-cell lymphoma-extra large
BRAF: serine/threonine protein kinase B-Raf
CoA: coenzyme A
COVID-19: coronavirus disease 2019
CPS1: carbamoylphosphate synthetase I
CPT1A: carnitine palmitoyl transferase 1A
CRC: colorectal cancer
DRP1: dynamin-related protein 1
E2F1: E2 transcription factor 1
ECHA: enoyl-CoA hydratase
ETC: electron transport chain
EDG: electron-donating group
EWG: electron-withdrawing group
FAO: fatty acid β-oxidation
FMO: flavin-containing monooxygenases
FOXO3a: Forkhead box O3a
G6PD: glucose-6-phosphate dehydrogenase
GAPDH: glyceraldehyde 3-phosphate dehydrogenase
GCN5: general control nondepressible 5
GLS: glutaminase
GLUD1: glutamate dehydrogenase 1
GOT: glutamic-oxaloacetic transaminase
GSH: reduced glutathione
GSR: glutathione reductase
GSSG: oxidized glutathione
HAT: histone acetyltransferase
HCC: hepatocellular carcinoma
HMGCS2: 3-hydroxy-3-methylglutaryl-CoA synthetase 2
HO-1: heme oxygenase 1
IDH: isocitrate dehydrogenase
KAT8: lysine acetyltransferase 8
*k*_cat_: catalytic constant
*K*_D_: dissociation constant
KO: knockout
KRAS: Kirsten rat sarcoma virus
LDH: lactate dehydrogenase
LINC: linker of nucleoskeleton and cytoskeleton
ME1: NADP-dependent malic enzyme
MEFs: mouse embryonic fibroblasts
MITF: microphthalmia-associated transcription factor
MPP^+^: 1-methyl-4-phenylpyridinium
MPTP: 1-methyl-4-phenyl-1,2,3,6-tetrahydropyridine
NAT10: *N*-acetyltransferase 10
NMT: *N*-terminal glycine myristoyltransferase
NRF2: nuclear factor erythroid 2-related factor
Nsp: nonstructural protein
OGDH: oxoglutarate dehydrogenase
PDAC: pancreatic ductal adenocarcinoma
PDC: pyruvate dehydrogenase complex
PGC-1α: peroxisome proliferator-activated receptor coactivator-1α
PKM2: pyruvate kinase M2
PTEN: phosphatase and tensin homologue
PTM: post-translational modification
ROS: reactive oxygen species
SARS-CoV-2: severe acute respiratory syndrome coronavirus 2
SDH: succinate dehydrogenase
SDHA: succinate dehydrogenase complex subunit A
SHMT2: serine hydroxymethyltransferase 2
Sir2: silent information regulator 2
SIRT: sirtuin
SOD: superoxide dismutase
STAT3: signal transducer and activator of transcription 3
SUN2: SAD1/UNC84 domain protein 2
TCA: tricarboxylic acid
TrxR2: thioredoxin reductase 2
THF: tetrahydrofolate
UCP-1: uncoupling protein 1
VDAC3: voltage-dependent anion channel 3
VLCAD: very long-chain acyl-CoA dehydrogenase
WAT: white adipose tissue
